# Supplementary material for: The association of presbyphagia with urinary incontinence and Incontinence-related quality of life in nursing home residents: A cross-sectional study
Source: PLoS One. 2026 Jun 4;21(6):e0350933. doi: 10.1371/journal.pone.0350933 (PMC13235875; doi:10.1371/journal.pone.0350933)
Supplement: Attachment — Submitted filename: Reviewed_PONE-D-25-62171_R1.pdf [file pone.0350933.s002.pdf]

PLOS One

The Association of Presbyphagia with Urinary Incontinence and Incontinence-Related Quality of Life in Nursing Home Residents: A Cross-Sectional Study

--Manuscript Draft--

**Manuscript Number:**	PONE-D-25-62171R1
**Article Type:**	Research Article
**Full Title:**	The Association of Presbyphagia with Urinary Incontinence and Incontinence-Related Quality of Life in Nursing Home Residents: A Cross-Sectional Study
**Short Title:**	Presbyphagia in the elderly
**Corresponding Author:**	Ziya Yıldız<br>Isparta Uygulamali Bilimler Universitesi<br>Uluborlu, Isparta TÜRKIYE
**Keywords:**	Swallowing disorder; Oral frailty; Presbyphagia; urinary incontinence; Incontinence-related quality of life
**Abstract:**	Purpose: The association between presbyphagia (age-related swallowing decline) and urinary incontinence (UI) remains poorly understood. This study therefore investigated the relationship between presbyphagia and both UI and incontinence-specific quality of life (I-QOL) in a distinct population of functionally independent healthy elderly in nursing home.<br>Method: The presence of presbyphagia was determined in 73 participants in the nursing home using the 100ml water swallowing test (100mlSwal) and the Eating Assessment Tool-10 (EAT-10) tests. UI was determined using the International Consultation on Incontinence Questionnaire Short (ICIQ-SF) and I-QOL was determined using the King's Health Questionnaire (KHQ). The presence of presbyphagia, age and female gender confounding factors were analysed by multiple regression analysis. Multiple linear and binary logistic regression analyses were performed with presbyphagia, age, gender, and handgrip strength. Binary logistic regression was used to calculate odds ratios for UI presence.<br>Results: Presbyphagia was significantly associated with UI (p=.001). Individuals with presbyphagia had 7.47-fold higher odds of UI. Presbyphagia was also significantly associated with multiple I-QOL sub-domains, including incontinence impact, role limitation, physical limitation, and emotions (all p<.001). Male gender was associated with higher UI presence (OR=5.16), but not with UI severity. Age showed no significant associations with UI or I-QOL.<br>Conclusion: Presbyphagia is independently associated with UI presence and poorer I-QOL in nursing home residents. Male gender was associated with higher UI prevalence but similar severity and I-QOL to females. These findings suggest an independent association between presbyphagia and UI, thereby contributing to the formulation of hypotheses aimed at understanding the complex interactions between oral frailty and the urinary system. Further research is needed to elucidate underlying mechanisms and explore the potential clinical implications of this association.
**Order of Authors:**	Ziya Yıldız
**Response to Reviewers:**	Response to Reviewer #1<br><br>Reviewer #1: “…However, there is a central point that deserves greater attention: presbyphagia and urinary incontinence frequently coexist because both emerge from the same pathophysiological context. This shared set of determinants creates a scenario in which any observed association between the two conditions is likely explained by confounding factors, especially sarcopenia. Since this important confounder was not analysed, even statistical adjustments cannot fully address this limitation, which compromises the interpretation of the results. Therefore, I recommend restructuring the text so that the findings related to the dependent variables—presbyphagia and urinary incontinence—are presented in relation to quality-of-life measures independently, avoiding causal inferences between presbyphagia and incontinence.”<br>Answer: Thank you for your insightful comment regarding the shared

Powered by Editorial Manager® and ProduXion Manager® from Aries Systems Corporation

	pathophysiological background of presbyphagia and urinary incontinence, and the potential confounding role of sarcopenia. You have raised a very important point that we had not initially considered. In response to your valuable feedback, we recognized the need to control for sarcopenia in our analysis. Since handgrip strength measurements are routinely collected in our institution every three months and recorded in the institutional database, we obtained the values corresponding to the study period and attempted to incorporate them into our regression model as a proxy measure for sarcopenia. However, during the diagnostic process for multicollinearity, we encountered a critical statistical issue: a very high correlation between handgrip strength and gender (r = -.959) (Figure 1), resulting in Variance Inflation Factor (VIF) values of 12.55 and 12.54 (Figure 2), respectively. Since VIF values significantly exceeded the recommended threshold of 5 (Craney and Surles 2002), including both variables would have compromised the reliability and validity of our model. Consequently, despite our attempt to address your concern by obtaining and testing handgrip strength data, we had to exclude it from the final analysis due to these statistical limitations.<br>As noted by O'Brien (2007), when VIF values exceed recommended thresholds, researchers commonly address multicollinearity by removing one of the correlated variables, using ridge regression, or combining variables into a single index. Similarly, Kim (2019) emphasizes that excluding multicollinear predictors is an appropriate approach to obtain stable and reliable regression models. Following these established methodological recommendations, we chose to exclude handgrip strength from our analyses, as its high correlation with gender (r = -.959; VIF > 5) made simultaneous inclusion of both variables statistically problematic. This decision ensured the consistency and reliability of our regression models. As you recommended, we have now explicitly stated in the Limitations section that, although we attempted to include handgrip strength, sarcopenia could not be adequately controlled for as a potential confounder. Due to these changes and to avoid causal inferences, we have restructured the text.<br>Figure 1. Correlations (handgrip vs gender) (The Figure file format is not supported, so it could not be added here. The Figure can be viewed in the relevant Word document.)<br>Figure 2. VIF value (The Figure file format is not supported, so it could not be added here. The Figure can be viewed in the relevant Word document.)<br>Reference:<br>Craney, T. A., & Surles, J. G. (2002). Model-Dependent Variance Inflation Factor Cutoff Values. Quality Engineering, 14(3), 391–403. https://doi.org/10.1081/QEN-120001878<br>O’brien, R.M. A Caution Regarding Rules of Thumb for Variance Inflation Factors. Qual Quant 41, 673–690 (2007). https://doi.org/10.1007/s11135-006-9018-6<br>Kim, J. H. (2019). Multicollinearity and misleading statistical results. Korean journal of anesthesiology, 72(6), 558-569.<br><br>Response to Reviewer #2<br>Reviewer #2: “The abstract is slightly long and statistically dense. Abstract could be made little simpler..”<br>Answer: Thank you for your valuable feedback. We have revised the abstract to be more concise and less statistically dense. We believe this version is simpler and more accessible to readers.<br>Reviewer #2: “This is a cross-sectional study, but at several places the wording sounds like presbyphagia leads to urinary incontinence. Please be careful with language-Say “associated with”, not “predicts”, “affects”, or “should be screened as standard practice”. The conclusions and clinical suggestions should be more cautious and framed as hypothesis-generating, not practice-changing.”<br>Answer: a) Thank you for your insightful comment regarding the language used in our manuscript. You are absolutely right that in a cross-sectional study, terminology should reflect associations rather than causal relationships. Some of these wording issues arose during the translation process from our native language to English. We have now carefully revised the manuscript accordingly. For example:<br>Original: "Multiple linear analysis was performed to predict the UI dependent variables using the independent variable of presence/absence of presbyphagia, age and gender."<br>Revised: "Multiple linear regression analysis was conducted to examine the association between UI and the independent variables: presence/absence of

Powered by Editorial Manager® and ProduXion Manager® from Aries Systems Corporation

presbyphagia, age, and gender."
b) In response to your comment, we have carefully revised the conclusion and discussion section accordingly. The overall tone of the conclusion now reflects a more cautious and hypothesis-generating perspective, highlighting the need for future longitudinal and interventional studies to confirm our findings.
Reviewer #2: Definition of presbyphagia needs clearer justification
Answer: We have substantially revised the introduction to provide a clearer, more comprehensive definition and justification of presbyphagia. The revised text now:
•Provides a precise, evidence-based definition of presbyphagia (structural, physiological, and innervational alterations)
•Specifies the underlying mechanisms (brain atrophy, neurological deterioration, muscle loss)
•Maintains connection to clinical symptoms with appropriate citations
We believe this revision addresses the reviewer's concern by clearly justifying what presbyphagia is and how it develops.
Reviewer #2: statistical analysis: The logistic regression model used to calculate the odds ratio is not clearly described Confidence intervals for the odds ratio are missing. Clearly state which analysis was used for the odds ratio. Report adjusted OR with 95% CI. Be consistent in how UI is analysed and interpreted
Answer: We thank the reviewers for their valuable comments regarding the odds ratio calculation and presentation. We have carefully addressed these points as follows:
•Removal of unclear odds ratio: The previous version of the manuscript included a statement: *'Participants were classified as having/not having UI according to the ICIQ-SF cut-off value and an odds ratio of 4.61 was calculated.'* We agree with the reviewers that this statement was insufficiently detailed, lacking information about the statistical method used, confidence intervals, and adjustment for confounders. This unclear statement has been removed entirely from the revised manuscript.
•Proper logistic regression analysis: In its place, we have now conducted and reported a binary logistic regression analysis following standard methodological guidelines. In the Statistical Analysis section, we clearly describe:
Variable coding: presbyphagia (0 = non-presbyphagia, 1 = presbyphagia), UI presence (ICIQ-SF ≥6; 0 = no UI, 1 = UI present)
Covariates: age and gender
Exclusion of handgrip strength due to high multicollinearity with gender (VIF > 5, r = -0.96)
•Complete reporting of results: In the Results section, we now present the adjusted odds ratio with its 95% confidence interval: OR = 7.47 (95% CI: 2.24–24.94, p = 0.001). A detailed table (Table 3) following simplified APA format now includes B coefficients, standard errors, Wald statistics, p-values, odds ratios, and confidence intervals for all variables. Model fit statistics ($\chi^2 = 21.53$, df = 3, $p < 0.001$; Cox & Snell $R^2 = 0.26$; Nagelkerke $R^2 = 0.35$; correct classification = 71.2%) are also reported.
•Addressing confidence interval width: We acknowledge that the confidence interval (2.24–24.94) is relatively wide. This limitation is now addressed in the Discussion section, where we note that the wide CI likely reflects the relatively small sample size and the exploratory nature of this study. Importantly, the lower bound remains substantially above 1 (2.24), supporting the robustness of the finding.
Reviewer #2: Figure 1 needs a clearer explanation in the text
Answer: "Thank you for your comment regarding Figure 1. We have revised the manuscript to provide a clearer explanation of the figure in the text.
In the revised Results section, we now state:
'Based on the ICIQ-SF question "When does urine leak?", 16.44% of participants (n=12) reported no urine leakage. Among the 83.56% of participants who experienced some form of incontinence, the most frequently reported circumstances were: leakage when asleep (20.55%, n=15), leakage when coughing or sneezing (17.81%, n=13), leakage during physical activity (15.07%, n=11), leakage after finishing urination (9.59%, n=7), leakage before reaching the toilet (8.22%, n=6), constant leakage (6.85%, n=5), and leakage for no obvious reason (5.48%, n=4). The distribution of urinary incontinence patterns is illustrated in Figure 1.'
Additionally, we have expanded the figure caption to: *'Distribution of urinary incontinence patterns among nursing home residents (n=73). Based on ICIQ-SF item: "When does urine leak?" Values represent percentage of the total sample and number of participants.'*
We believe these revisions provide a clearer and more comprehensive explanation of Figure 1 as requested."

Powered by Editorial Manager® and ProduXion Manager® from Aries Systems Corporation

Reviewer #2: Tables are very dense and heavy on abbreviations
Answer: We have revised all tables to improve clarity and reduce density. Simplified table footnotes by removing redundant abbreviations and providing only essential information in a clear, concise format. Combined the unstandardized regression coefficient (B) and its 95% confidence interval into a single column (B [95% CI]) to streamline presentation and enhance readability.
Reviewer #2: UI and swallowing function can also be influenced by:BMI, Mobility, Comorbidities, Hydration status, Medication burden.If these variables are unavailable, please explicitly acknowledge residual confounding as a limitation. If some data exist (e.g., BMI), consider including them in an exploratory model.
Answer:
•Handgrip strength (sarcopenia): In response to reviewer comments regarding potential confounding by generalized muscle weakness and sarcopenia, we obtained handgrip strength data from institutional records—as these measurements are routinely collected every three months in our nursing home—and attempted to include this variable in our regression models as a proxy for sarcopenia. However, during diagnostic testing for multicollinearity, we encountered a significant statistical issue. The correlation matrix revealed an extremely high negative correlation between handgrip strength and gender ($r = -.959$), indicating that these two variables shared almost identical variance. Consequently, the Variance Inflation Factor (VIF) values for gender and handgrip strength were 12.55 and 12.54, respectively—far exceeding the recommended threshold of 5 [Craney and Surles 2002; O'brien 2007]. As noted by Craney and Surles (2002), VIF values above 5 indicate problematic multicollinearity that can distort regression coefficients, inflate standard errors, and compromise the reliability and validity of statistical models. Following established methodological recommendations [Kim 2009], researchers facing high multicollinearity have several options: remove one of the correlated variables, use ridge regression, or combine variables into a single index. Given that gender is a fundamental demographic variable with strong theoretical justification for inclusion, and given the exceptionally high correlation between handgrip strength and gender, we made the considered decision to exclude handgrip strength from the final models. This approach ensures stable, interpretable, and reliable regression results. We have now explicitly acknowledged in the Limitations section that, despite our attempt to include handgrip strength, sarcopenia could not be fully controlled for as a potential confounder due to these statistical constraints.
Reference: Craney, T. A., & Surles, J. G. (2002). Model-Dependent Variance Inflation Factor Cutoff Values. Quality Engineering, 14(3), 391–403. https://doi.org/10.1081/QEN-120001878
O'brien, R.M. A Caution Regarding Rules of Thumb for Variance Inflation Factors. Qual Quant 41, 673–690 (2007). https://doi.org/10.1007/s11135-006-9018-6
Kim, J. H. (2019). Multicollinearity and misleading statistical results. Korean journal of anesthesiology, 72(6), 558-569.
Figure 1. Correlations (handgrip vs gender) (The Figure file format is not supported, so it could not be added here. The Figure can be viewed in the relevant Word document.)
Figure 2. VIF value (The Figure file format is not supported, so it could not be added here. The Figure can be viewed in the relevant Word document.)
•BMI: We chose not to include BMI in the final models for several methodological reasons. First, to avoid increasing Type II error risk: Adding BMI as an additional covariate would increase the number of statistical tests, thereby lowering the significance threshold when applying multiple comparison corrections (e.g., Bonferroni). This could obscure truly meaningful associations. Second, with a sample size of 73, including too many covariates risks overfitting and unstable estimates (Pergener, 1998). Following principles of parsimonious modeling, we prioritized variables with stronger theoretical justification: presbyphagia, age, and gender. Third, preliminary bivariate analyses showed no significant association between BMI and our primary outcomes, further supporting this decision. We believe this balanced approach maintains statistical power while appropriately addressing confounding.
Referance: "Perneger TV. What's wrong with Bonferroni adjustments. BMJ. 1998 Apr 18;316(7139):1236-8. doi: 10.1136/bmj.316.7139.1236. PMID: 9553006; PMCID: PMC1112991.
•Mobility, comorbidities, and medication burden: These potential confounders were addressed through rigorous a priori exclusion criteria, which we believe strengthens our methodological approach.
Mobility: Individuals with a history of falls or requiring ambulation assistance were

excluded (criterion #2), ensuring a functionally independent sample with preserved mobility.
Comorbidities: We excluded individuals with neurological/psychiatric diseases (criterion #3), conditions affecting swallowing/UI mechanisms (criterion #4), and acute systemic illnesses (criterion #6), thereby reducing confounding from major comorbidities.
Medication burden: Regular use of medications affecting urinary function or cognition (e.g., diuretics, anticholinergics) was an exclusion criterion (criterion #5), eliminating pharmacological confounding from these classes.
•Hydration : We acknowledge that hydration status was not directly measured in this study, and we have now added this as an explicit limitation in the revised manuscript. Future studies should incorporate objective measures of hydration status to further clarify its potential role in the presbyphagia-UI relationship.

Response to Reviewer #3

Reviewer #3: This is an original study that raises an interesting but conceptually challenging question. The primary aim is to demonstrate an association between presbyphagia and urinary incontinence. While such an association is statistically demonstrated, causation is not established, and the clinical or practical implications of this finding remain unclear. The results are statistically significant but conceptually weak in terms of mechanistic or clinical interpretation.
Answer: We thank the reviewer for these important observations regarding causal inference and clinical interpretation. We fully agree that this cross-sectional study cannot establish causality, and we have carefully revised the manuscript throughout to reflect this.
Specifically, we have: Replaced all causal language (e.g., "predicts," "leads to") with associative terms ("is associated with," "is linked to") throughout the manuscript. Softened all clinical implications, presenting findings as hypothesis-generating rather than practice-changing. For example, statements suggesting routine screening have been revised to indicate that presbyphagia "may warrant clinical attention" in comprehensive geriatric assessment. Added discussion of potential mechanisms (e.g., autonomic nervous system dysfunction, shared neuromuscular pathways) while explicitly noting that these remain speculative and require confirmation in future studies.
Reviewer #3: “Several methodological concerns merit attention. Urinary incontinence is not a single clinical entity; stress and urgency incontinence have distinct mechanisms and etiologies. In the present study, incontinence appears to be treated as a unified condition and linked to presbyphagia as a single construct, which may oversimplify the clinical reality.”
Answer: We thank the reviewer for this important observation. While our primary regression analyses treated UI as a unified condition (using ICIQ-SF total scores), we did collect data on UI subtypes through the ICIQ-SF item "When does urine leak?" As presented in Figure 1, the distribution was: stress-pattern UI (cough/sneeze + physical activity) in 24 participants (32.9%), urgency-pattern UI in 6 participants (8.2%), other patterns in 31 participants (42.5%), and no leakage in 12 participants (16.4%). Due to the small number of participants with urgency UI (n=6), subtype-specific statistical analyses were not feasible. We have now acknowledged this as a limitation (Limitations section, last point) and recommend that future studies with larger samples investigate subtype-specific associations.
Reviewer #3: In addition, the sample size is small and drawn from a single population, limiting generalisability.
Answer: We fully concur with the reviewer that the sample size (n=73) is modest and drawn from a single population, which limits generalizability. However, it is important to note that this final sample was derived from an initial pool of 298 residents after applying rigorous exclusion criteria to ensure a homogeneous, functionally independent population without major confounders. A post-hoc power analysis using the observed effect size ($f^2 = 0.17$) from our study data confirmed that the study achieved 97% statistical power at a 0.05 significance level. This indicates that despite the modest sample size and the effect size being lower than initially anticipated, our sample was adequately powered to detect the observed associations, supporting the

Powered by Editorial Manager® and ProduXion Manager® from Aries Systems Corporation

internal validity of the findings.
We have revised the manuscript throughout to emphasize the exploratory, hypothesis-generating nature of this study. The Limitations section now explicitly acknowledges the limited generalizability due to the single-center design and modest sample size, while the Conclusion emphasizes that our study provides a foundation for future research with larger, more diverse cohorts rather than definitive clinical recommendations.
Reviewer #3: Overall, the study is best interpreted as exploratory and association-based, rather than as a hypothesis-confirming or clinically directive investigation.
Answer: We fully concur with the reviewer. We have revised the manuscript throughout to emphasize the exploratory, hypothesis-generating nature of this study. All causal language has been replaced with associative terminology, and clinical implications have been softened to reflect the preliminary nature of the findings. The Conclusion now explicitly states that our study provides a foundation for future research rather than definitive clinical recommendations.. We have carefully reviewed the entire manuscript and replaced all instances of causal language (e.g., "predicts," "affects," "leads to") with appropriate associative terms such as "is associated with," "is related to," and "is linked to" throughout the Abstract, Results, Discussion, and Conclusion sections.
Reviewer #3: “The main strengths of the manuscript include the use of validated questionnaires, clear reporting of regression coefficients and correlations, and the absence of obvious internal data inconsistencies. However, it would be more appropriate to replace the term “predicts” with “is associated with” throughout the manuscript when describing regression results.”
Answer: Thank you for your positive feedback regarding the use of validated questionnaires and clear reporting of statistical results. We appreciate your constructive suggestion regarding terminology. In response, we have carefully reviewed the entire manuscript and replaced all instances of causal language (e.g., "predicts," "affects," "leads to") with appropriate associative terms such as "is associated with," "is related to," and "is linked to" throughout the text. We believe this revision more accurately reflects the cross-sectional nature of our study and aligns with your recommendation. Thank you for helping us improve the precision of our language.
Reviwer 3: “Although I am not a statistician, the manuscript would benefit from formal statistical review. In particular, there appears to be a multiple comparisons issue, with approximately 30 hypothesis tests conducted without correction.”
Answer: “We have carefully considered this and implemented a balanced, theoretically justified approach:
•For the 10 KHQ subscales: These represent a family of related outcomes measuring the same underlying construct (incontinence-specific quality of life). We applied Bonferroni correction within this domain only ($\alpha = 0.05/10 = 0.005$).
•For the ICIQ-SF (UI) analysis: This tests a separate, independent hypothesis (presbyphagia-UI association) distinct from the QOL outcomes. Applying Bonferroni across unrelated research questions would be excessively conservative and increase Type II error risk, potentially obscuring real associations (Perneger, 1998; Rothman, 1990). Therefore, we did not apply correction to this primary hypothesis test.
•We have added a short sentence to the Statistical Analysis section to address this correction.
We believe this nuanced approach appropriately balances the risks of both Type I and Type II eros while maintaining scientific rigor.”
Referance: “Perneger TV. What's wrong with Bonferroni adjustments. BMJ. 1998 Apr 18;316(7139):1236-8. doi: 10.1136/bmj.316.7139.1236. PMID: 9553006; PMCID: PMC1112991.”
Reviwer 3: “Additionally, a logistic regression analysis is implied by the reporting of an odds ratio (OR = 4.61), but the corresponding model and confidence intervals are not presented.”
Answer: In the revised manuscript, we have now clearly described the binary logistic regression analysis in the Statistical Analysis section and presented the adjusted odds ratios with their 95% confidence intervals. For the association between presbyphagia and UI, the adjusted odds ratio is AOR = 7.47 (95% CI: 2.24–24.94, p = 0.001) , adjusted for age and gender. Additionally, male gender was independently associated with higher odds of UI presence (AOR = 5.16, 95% CI: 1.53–17.42, p = 0.008) . The initial OR value of 4.61 was a preliminary estimate and has been replaced with these more robust analyses. We have also added a new table presenting the full binary logistic regression results, including all covariates and their corresponding odds ratios, confidence intervals, and p-values.

Powered by Editorial Manager® and ProduXion Manager® from Aries Systems Corporation

	Reviwer 3: Conceptual overlap: Presbyphagia is defined using EAT-10, a symptom-based instrument, while outcomes include multiple symptom-based and quality-of-life scales (ICIQ-SF, KHQ). The observed associations may therefore reflect overall symptom burden, frailty, or response bias rather than a specific link between swallowing function and urinary continence. This limitation should be explicitly acknowledged, and conclusions reframed accordingly.<br>Answer: We acknowledge that both presbyphagia and UI outcomes were assessed using symptom-based questionnaires. Therefore, the observed associations may partially reflect overall symptom burden or response bias rather than a specific physiological link.<br>In response to this valuable feedback, we have:<br>•Expanded our existing limitations statement (formerly "Third, self-reported data are susceptible to recall and response biases") to now explicitly read: "Third, self-reported data are susceptible to recall and response biases, as both presbyphagia and UI outcomes were assessed using symptom-based questionnaires."<br>•Added a corresponding statement in the Conclusion section to ensure interpretation reflects this limitation: "Additionally, both presbyphagia and UI outcomes were assessed using symptom-based questionnaires, which introduces the possibility that the observed associations may partially reflect overall symptom burden or response bias rather than a specific physiological link."<br>These revisions ensure that this limitation is both explicitly acknowledged and consistently reflected in our interpretation of the findings. We believe this addresses the reviewer's concern appropriately.<br>Reviwer 3: Causality: At several points, the manuscript implies a directional or causal relationship. These statements should be revised to consistently emphasise association only.<br>Answer: In response, we have carefully reviewed the entire manuscript and replaced all causal language (e.g., "predicts," "affects," "leads to") with appropriate associative terms such as "is associated with," "is related to," and "is linked to" throughout the Abstract, Results, Discussion, and Conclusion sections. The manuscript now consistently emphasizes association rather than causation.<br>Reviwer 3: Clinical relevance: The variance explained by the regression models is modest (R^2 approximately 0.10–0.20). Statements suggesting clinical screening, diagnostic implications, or practice change should therefore be avoided.<br>Answer: We have softened all clinical implications throughout the manuscript. Statements suggesting clinical screening or practice change have been revised to reflect the exploratory nature of our findings. The Conclusion now emphasizes that our study provides a foundation for future research rather than definitive clinical recommendations.<br>Reviwer 3: If available, comorbidity or frailty measures should be considered as potential confounders.<br>Answer: Regarding comorbidities, our study design addressed this through rigorous a priori exclusion criteria. Participants with known neurological or psychiatric diseases (e.g., stroke, Parkinson's disease, dementia), conditions directly affecting swallowing or UI mechanisms, and acute or uncontrolled systemic illnesses were excluded from the study. This approach resulted in the exclusion of 225 out of 298 initially assessed individuals, yielding a final sample of 73 functionally independent older adults with minimal major comorbidities. We obtained handgrip strength measurements—which are routinely collected in our institution every three months—from the institutional database and attempted to incorporate them into our regression model as a proxy measure for sarcopenia. However, during diagnostic testing for multicollinearity, we encountered a critical statistical issue: an extremely high correlation between handgrip strength and gender ($r = -.959$), resulting in Variance Inflation Factor (VIF) values of 12.55 and 12.54, respectively. These values substantially exceeded the recommended threshold of 5 (Craney and Surles 2002; O'Brien 2007), indicating that including both variables would have compromised the reliability and validity of our model.<br>Following established methodological recommendations (Kim 2019), we chose to exclude handgrip strength from our analyses, as its high correlation with gender made simultaneous inclusion statistically problematic. This decision ensured the consistency and reliability of our regression models. As you recommended, we have now explicitly stated in the Limitations section that, despite our attempt to include handgrip strength, sarcopenia could not be adequately controlled for as a potential confounder.<br>Figure 1. Correlations (handgrip vs gender) (The Figure file format is not supported, so it could not be added here. The Figure can be viewed in the relevant Word document.)

Powered by Editorial Manager® and ProduXion Manager® from Aries Systems Corporation

	Figure 2. VIF value (The Figure file format is not supported, so it could not be added here. The Figure can be viewed in the relevant Word document.)<br>Reference:<br>Craney, T. A., & Surles, J. G. (2002). Model-Dependent Variance Inflation Factor Cutoff Values. Quality Engineering, 14(3), 391–403. https://doi.org/10.1081/QEN-120001878<br>O'brien, R.M. A Caution Regarding Rules of Thumb for Variance Inflation Factors. Qual Quant 41, 673–690 (2007). https://doi.org/10.1007/s11135-006-9018-6<br>Kim, J. H. (2019). Multicollinearity and misleading statistical results. Korean journal of anesthesiology, 72(6), 558-569.<br>Reviwer 3: Recruitment setting and exclusion criteria should be clarified.<br>Answer: The recruitment setting and exclusion criteria are already detailed in the Methods section under "2.1 Study design and setting" and "2.2 Participants and Sample Size". As described, participants were recruited from two nursing homes in Isparta, Turkey, between December 23-30, 2024. A total of 298 residents were assessed for eligibility, and 225 were excluded based on the predefined exclusion criteria listed in the manuscript. Although we recorded the exact numbers excluded for each criterion, we chose not to include these detailed frequencies in the manuscript to avoid lengthening the text unnecessarily. The remaining 73 participants constituted the final sample. We believe this provides a comprehensive and transparent description of the participant selection process. If the editor or reviewers feel that including these detailed exclusion numbers would strengthen the manuscript, we are happy to add them.<br>Reviwer 3: In conclusion, the study demonstrates a statistical association between presbyphagia and urinary incontinence. Older adults classified as having presbyphagia score worse on ICIQ-SF, KHQ, and quality-of-life questionnaires. These findings are compatible with broader constructs such as frailty, comorbidity, and symptom burden. However, the study does not establish a causal relationship, does not identify a shared physiological mechanism, and does not justify clinical screening for urinary incontinence based on presbyphagia alone. The manuscript may be considered for publication provided the above issues are adequately addressed and the exploratory nature of the findings is clearly acknowledged.<br>Answer: We fully agree that this study demonstrates a statistical association rather than a causal relationship. In response, we have emphasized the exploratory nature of our findings throughout the manuscript, replaced all causal language with associative terminology, softened clinical implications, and expanded the Limitations section to address the issues raised. We believe these revisions adequately address the reviewer's concerns.
**Additional Information:**	
**Question**	**Response**
**Financial Disclosure**<br><br>Enter a financial disclosure statement that describes the sources of funding for the work included in this submission. Review the submission guidelines for detailed requirements. View published research articles from *PLOS ONE* for specific examples.<br><br>This statement is required for submission and **will appear in the published article** if the submission is accepted. Please make sure it is accurate.	The author(s) received no specific funding for this work.

**Funded studies**<br>Enter a statement with the following details:<br>• Initials of the authors who received each award<br>• Grant numbers awarded to each author<br>• The full name of each funder<br>• URL of each funder website<br>• Did the sponsors or funders play any role in the study design, data collection and analysis, decision to publish, or preparation of the manuscript?<br><br>Did you receive funding for this work?	
**Competing Interests**<br><br>Use the instructions below to enter a competing interest statement for this submission. On behalf of all authors, disclose any competing interests that could be perceived to bias this work—acknowledging all financial support and any other relevant financial or non-financial competing interests.<br><br>This statement is required for submission and **will appear in the published article** if the submission is accepted. Please make sure it is accurate and that any funding sources listed in your Funding Information later in the submission form are also declared in your Financial Disclosure statement.<br><br>View published research articles from *PLOS ONE* for specific examples.	The authors have declared that no competing interests exist.

Powered by Editorial Manager® and ProduXion Manager® from Aries Systems Corporation

**NO authors have competing interests**<br><br>Enter: *The authors have declared that no competing interests exist.*<br><br>**Authors with competing interests**<br><br>Enter competing interest details beginning with this statement:<br><br>*I have read the journal's policy and the authors of this manuscript have the following competing interests: [insert competing interests here]*<br><br>* typeset	
**Ethics Statement**<br><br>Enter an ethics statement for this submission. This statement is required if the study involved:<br><br>• Human participants<br>• Human specimens or tissue<br>• Vertebrate animals or cephalopods<br>• Vertebrate embryos or tissues<br>• Field research<br><br>Write "N/A" if the submission does not require an ethics statement.<br><br>General guidance is provided below. Consult the submission guidelines for detailed instructions. **Make sure that all information entered here is included in the Methods section of the manuscript.**	The study was approved by the Research Ethics Committee of Isparta University of Applied Sciences on November 4, 2024 (CASE: E-96714346-050.04-139027, evaluation number: 203/5).

Powered by Editorial Manager® and ProduXion Manager® from Aries Systems Corporation

**Format for specific study types**<br><br>**Human Subject Research (involving human participants and/or tissue)**<br>• Give the name of the institutional review board or ethics committee that approved the study<br>• Include the approval number and/or a statement indicating approval of this research<br>• Indicate the form of consent obtained (written/oral) or the reason that consent was not obtained (e.g. the data were analyzed anonymously)<br><br>**Animal Research (involving vertebrate animals, embryos or tissues)**<br>• Provide the name of the Institutional Animal Care and Use Committee (IACUC) or other relevant ethics board that reviewed the study protocol, and indicate whether they approved this research or granted a formal waiver of ethical approval<br>• Include an approval number if one was obtained<br>• If the study involved *non-human primates*, add *additional details* about animal welfare and steps taken to ameliorate suffering<br>• If anesthesia, euthanasia, or any kind of animal sacrifice is part of the study, include briefly which substances and/or methods were applied<br><br>**Field Research**<br><br>Include the following details if this study involves the collection of plant, animal, or other materials from a natural setting:<br>• Field permit number<br>• Name of the institution or relevant body that granted permission	
**Data Availability**<br><br>Authors are required to make all data underlying the findings described fully available, without restriction, and from the time of publication. PLOS allows rare exceptions to address legal and ethical concerns. See the PLOS Data Policy and FAQ for detailed information.	Yes - all data are fully available without restriction

A Data Availability Statement describing where the data can be found is required at submission. Your answers to this question constitute the Data Availability Statement and **will be published in the article**, if accepted.<br><br>**Important:** Stating 'data available on request from the author' is not sufficient. If your data are only available upon request, select 'No' for the first question and explain your exceptional situation in the text box.<br><br>Do the authors confirm that all data underlying the findings described in their manuscript are fully available without restriction?	
**Describe where the data may be found in full sentences. If you are copying our sample text, replace any instances of XXX with the appropriate details.**<br><br>• If the data are **held or will be held in a public repository**, include URLs, accession numbers or DOIs. If this information will only be available after acceptance, indicate this by ticking the box below. For example: *All XXX files are available from the XXX database (accession number(s) XXX, XXX.)*.<br>• If the data are all contained **within the manuscript and/or Supporting Information files**, enter the following: *All relevant data are within the manuscript and its Supporting Information files.*<br>• If neither of these applies but you are able to provide **details of access elsewhere**, with or without limitations, please do so. For example:<br><br>*Data cannot be shared publicly because of [XXX]. Data are available from the XXX Institutional Data Access / Ethics Committee (contact via XXX) for researchers who meet the criteria for access to confidential data.*<br><br>*The data underlying the results presented in the study are available from (include the name of the third party*	Data is shared via the Mendeley data system. https://data.mendeley.com/preview/s9z78b585n?a=3cb7ca47-c4fc-46f3-bf99-d5098fc7f55d<br><br>(The dataset is accessible via Mendeley Data. A free user account is required to log in and view the data, but registration is open to anyone and completely free of charge. There are no fees or any other restrictions for access. This complies with the PLOS ONE policy that requiring user registration is acceptable as long as anyone can register.)

and contact information or URL).<br>• This text is appropriate if the data are owned by a third party and authors do not have permission to share the data.<br><br>* typeset	
Additional data availability information:	Tick here if the URLs/accession numbers/DOIs will be available only after acceptance of the manuscript for publication so that we can ensure their inclusion before publication.

Powered by Editorial Manager® and ProduXion Manager® from Aries Systems Corporation

Manuscript Click here to access/download;Manuscript;Manuscript.docx

The Association of Presbyphagia with Urinary Incontinence and Incontinence-Related Quality of Life in Nursing Home Residents: A Cross-Sectional Study

Presbyphagia in the elderly

Ziya YILDIZ[1*]

[1*] PhD, Department of Physical Therapy and Rehabilitation, Uluborlu Vocational School, Isparta University of Applied Sciences, 32050, Isparta, TURKEY
Telephone: +90 507 507 7573

***Correspondence:** ziyayildiz@isparta.edu.tr

ORCID: https://orcid.org/0000-0001-6961-8202

Abstract

Purpose: The association between presbyphagia (age-related swallowing decline) and urinary incontinence (UI) remains poorly understood. This study therefore investigated the relationship between presbyphagia and both UI and incontinence-specific quality of life (I-QOL) in a distinct population of functionally independent healthy elderly in nursing home.

Method: The presence of presbyphagia was determined in 73 participants in the nursing home using the 100ml water swallowing test (100mlSwal) and the Eating Assessment Tool-10 (EAT-10) tests. UI was determined using the International Consultation on Incontinence Questionnaire Short (ICIQ-SF) and I-QOL was determined using the King's Health Questionnaire (KHQ). The presence of presbyphagia, age and female gender confounding factors were analysed by multiple regression analysis. Multiple linear and binary logistic regression analyses were performed with presbyphagia, age, gender, and handgrip strength. Binary logistic regression was used to calculate odds ratios for UI presence.

Results: Presbyphagia was significantly associated with UI (p=.001). Individuals with presbyphagia had 7.47-fold higher odds of UI. Presbyphagia was also significantly associated with multiple I-QOL sub-domains, including incontinence impact, role limitation, physical limitation, and emotions (all p<.001). Male gender was associated with higher UI presence

(OR=5.16), but not with UI severity. Age showed no significant associations with UI or I-QOL.

Conclusion: Presbyphagia is independently associated with UI presence and poorer I-QOL in nursing home residents. Male gender was associated with higher UI prevalence but similar severity and I-QOL to females. These findings suggest an independent association between presbyphagia and UI, thereby contributing to the formulation of hypotheses aimed at understanding the complex interactions between oral frailty and the urinary system. Further research is needed to elucidate underlying mechanisms and explore the potential clinical implications of this association.

Keywords: Swallowing disorder, Oral frailty, Presbyphagia, Urinary incontinence, Incontinence-related quality of life

***The clinical trials number of the study is NCT06827561.

1. INTRODUCTİON

UI and swallowing disorder (SD) are common problems in healthy elderly individuals living in nursing homes. One such SD is presbyphagia, defined as the structural, physiological, and innervational alterations in the swallowing process that occur as a result of healthy aging. These age-related changes—including progressive brain atrophy, neurological deterioration, and loss of muscle mass and strength in the oropharyngeal region—can lead to clinical symptoms such as choking, coughing, pharyngeal residue, aspiration pneumonia and impaired swallowing rate (1, 2). Presbyphagia is an overlooked health problem in the elderly because it is perceived as part of the normal aging process and develops secretly (3). It is the primary contributing factor to aspiration pneumonia in 50-75% of nursing home residents and is more common in this population compared to community-dwelling elderly (4).UI, which is considered a health priority by the World Health Organization, is another problem that emerges with aging (5). A multicentre study reported that the prevalence of UI in nursing homes was approximately 70% (6). UI, which is caused by weakness in the nerve and muscle structure (7), is an important cause of poor quality of life in the elderly in nursing homes (5). UI causes weaknesses such as decreased interpersonal relationships, loss of career and psychological well-being, limitations in the ability to exercise, and impaired sexual life (8).

The studies focusing UI and SD in the elderly have been associated with sarcopenia through different mechanisms (8, 9). However, this relationship between UI and sarcopenia in elderly patients with SD was not found (10). Theories such as the “soda pop model”, which explains the anatomical relationship between the pelvic floor, diaphragm and pharynx/larynx, attempt to theorize the complex interaction between UI and swallowing function (11). However, the literature explaining the relationship and mechanism between presbyphagia and UI and I-QOL remains limited. Although there is a study associating presbyphagia with lower urinary tract symptoms in middle-aged/elderly Japanese community-dwelling populations

(12), research focusing on elderly individuals in nursing homes, particularly in middle-income countries like Turkey where institutional residency often reflects socioeconomic status rather than health status, is notably absent. This population is particularly relevant given recent findings reporting a healthy ageing rate of 38.5% in Turkish nursing homes (13), indicating a substantial subgroup of healthy older adults in these settings.

In addition, effect modifiers and confounders associated with UI in nursing homes have not been adequately researched due to reasons such as facility characteristics, staffing issues, reimbursement and cultural attitudes (8). Although UI is known to be hidden behind multiple diseases, weakness or other geriatric syndromes, (14) its relationship with presbyphagia has not been fully established. Therefore, this study aimed to investigate the relationship between presbyphagia and UI and I-QOL in this novel population of healthy elderly nursing home residents. Within this context, we tested the hypothesis that UI and I-QOL would be significantly associated with presbyphagia.

2. METHOD

2.1 Study design and setting

This cross-sectional observational study was conducted between December 23-30, 2024, using clinical tests and a face-to-face interview survey instrument. Participants have provided written informed consent. The study included healthy elderly individuals aged 65-95 years at Gardenya Nursing Home and Hacı Ahmet Şençopur Nursing Home in Isparta, Turkey. The study was approved by the Research Ethics Committee of Isparta University of Applied Sciences on November 4, 2024 (CASE: E-96714346-050.04-139027, evaluation number: 203/5). The study was conducted in accordance with Declaration of Helsinki for research involving humans and the STROBE guidelines for conducting observational studies. Mini mental state examination was used to determine the mental status of the participants. After demographic characteristics of the volunteers were recorded, 100mlSwal and EAT-10

scale were used to determine SD, International Consultation on Incontinence Questionnaire Short Form (ICIQ-SF) was used to determine UI, and King's Health Questionnaire (KHQ) was used to determine the I-QOL.

2.2 Participants and Sample Size

The required sample size was calculated a priori using G*Power 3.1 software. For a multiple linear regression analysis with a significance level (α) of 0.05, a statistical power (1-β) of 0.95, and a high effect size ($f^2 = 0.35$), a minimum of 40 participants was required. The study initially assessed 298 elderly individuals from the nursing home for eligibility.

The inclusion criteria were: (1) being a resident of the nursing home for at least one year, (2) a Mini-Mental State Examination (MMSE) score of 24 or above, indicating no significant cognitive impairment, (3) the absence of advanced hearing or visual problems that would hinder communication, and (4) provision of voluntary informed consent.

The exclusion criteria were designed to ensure a sample of functionally independent older adults without conditions that could directly confound the study's outcomes. Individuals were excluded if they met any of the following criteria: (1) dependency in any of the Basic Activities of Daily Living as assessed by the Katz ADL Index (index point<6), (2) a history of falls within the past 6 months or requiring assistance for ambulation, (3) a known history of neurological or psychiatric diseases (e.g., stroke, Parkinson's disease, dementia, or major depressive disorder defined by a score >10 on the 15-item Geriatric Depression Scale), (4) a history of diseases or surgeries with a known direct impact on swallowing or UI mechanisms, (5) regular use of medications that could significantly affect lower urinary tract function or cognitive status (e.g., diuretics, anticholinergics, antipsychotics), (6) an acute or uncontrolled systemic illness (e.g., uncontrolled hypertension, decompensated heart failure), (7) a diagnosed urinary tract infection within the past 4 weeks.

Based on these criteria, 225 individuals were excluded. The final study sample consisted of 73 participants. A post-hoc power analysis was conducted using the observed effect size ($f^2 = 0.17$) from the study data. This analysis confirmed that the study achieved a high statistical power of 97% at a 0.05 significance level, thus validating the robustness of the findings despite the observed effect size being lower than the initially anticipated high effect size used for the a priori calculation.

2.3 Measurements

EAT-10 scale and 100mlSwal test were used in the evaluation of presbyphagia. The 100mlSwal test was measured by sitting the participants on a chair with armrests and asking them to drink water quickly and not to hold it in the mouth. The time until the thyroid cartilage returned to its resting position was recorded for participants who had completely finished the water. The swallowing rate (ml/s) was calculated for all participants by dividing the amount of water drunk by the time recorded. Abnormal swallowing rate was considered to be below 10 ml/s. (15). If the sum of the scores given to each item in the EAT-10 scale was 3 points or more, the risk of SD was accepted. Presbyphagia was considered to be present in healthy elderly individuals who scored 3 points or more on the EAT-10 scale (16) or who scored less than 10 ml/sec on the 100mlSwal test (15).

The ICIQ-SF is a scale assesses the frequency, severity in UI. A Likert scale is used to assess the presence/absence and severity of symptoms. The overall score range is 0 to 21, with higher values indicating increased symptom severity. The cut-off score to distinguish between those with and without UI is 6 (17).

KHQ was used to calculate the change in I-QOL. In the first part, Role, Physical, and Social Limitations, Limitations in Personal Relationship, Emotional Problems, Sleep and Energy Disturbances are evaluated. The second part includes the Symptom Severity Scale, which assesses the presence and severity of urinary symptoms (18).

2.4. Statistical Analysis

The data obtained were analysed using version 26.0 of the SPSS programme. Missing data were identified and corrected during data collection and entry. The skewness and kurtosis values of the data were checked for the assumption of normality, and the values were found to range between − 2.0 and + 2.0. The analysis was performed with presbyphagia/non-presbyphagia group, age, handgrip strength and gender as independent variables and UI and I-QOL as dependent variables. Multiple linear regression analysis was performed to explain the relationship between the variables. The R-squared (R^2) statistic evaluates the extent to which the regression model explains the variation in the dependent variable. The unstandardized beta coefficient (B) indicates the magnitude and direction of the relationship between the independent variables and the dependent variable. Pearson correlation and point-biserial correlation analysis was performed to determine the relationship between variables. Bonferroni correction was applied selectively to balance the risks of type 1 and type 2 errors. For the 10 KHQ subdimension—a coherent set of related outcomes assessing incontinence-specific quality of life—the significance threshold was set at $p < 0.005$ (0.05/10). The ICIQ-SF analysis for UI presence was considered a separate, independent hypothesis and was not subjected to multiple comparisons correction, consistent with standard practice for distinct research questions ($p<0.05$)Multivariable Binary logistic regression was used to assess the association between presbyphagia (0 = non-presbyphagia, 1 = presbyphagia) and UI presence (ICIQ-SF ≥6; 0 = no, 1 = yes), adjusting for age and gender. Handgrip strength was excluded from the regression models due to high multicollinearity with gender (r = -.959; VIF = 12.55 and 12.54 for gender and handgrip strength, respectively), which exceeded the recommended threshold of 5 and could compromise model stability (19). Results are presented as adjusted odds ratios (OR) with 95% confidence intervals (CI).

2.5. Validity and Reliability

The questionnaires were administered by a single researcher. The internal consistency of the Turkish EAT-10 scale was found to be 0.90 Cronbach's alpha and the test repeatability was 0.91 Cronbach's alpha (16). The ICIQ-SF is a scale with a validity level of A. The reliability and validity of the Turkish version was calculated as Cronbach's alpha value of 0.71 (17). The internal consistency (Cronbach's alpha value ≥0.68) and test-retest reliability of the Turkish version of the KHQ were found to be high (18).

3. RESULT

A total of 73 white elderly, 35 males and 38 females, participated in the study. The study included 32 participants with presbyphagia (16 males, 16 females) and 41 participants without presbyphagia (19 males, 22 females). The mean age of the participants was 76.53± 8.05 years. The mean body mass index was 24.08±1.2 kg/m^2. The mean swallowing rate was 15.4±5.32 ml/sec. Total ICIQ-SF score was 5.68±4.13 and Symptom Severity Scale was 6.45±5.92. The 100mlSwa test rate was 15.4± 5.32 ml/sn (Table 1). There were 32 participants diagnosed with presbyphagia, including 4 participants with a 100mlSwa test rate below 10 ml/sec, 19 participants with an EAT-10 score of 3 and above, and 9 participants who failed both tests.

Table 1. General information of the participants

	**n**	**Minimum**	**Maximum**	**X± SS**
Age (year)	73	65	92	76.5±8.05
ICIQTot	73	0	18	5.68±4.13
100mlSwal (ml/sn)	73	6.7	27	15.4±5.32
EAT-10	73	0	7	2.31±1.61
Handgrip strength	73	15.7	32.1	23.14±5.72
GeHea	73	0	75	30.47±22.53
InIm	73	0	100	21±24.53
RoLim	73	0	66.67	8.44±13.35
PhyLim	73	0	83.33	16.43±19.54
SoLim	73	0	50	14.1±15.7
Emo	73	0	88.89	19.39±25.32
SleEne	73	0	83.33	31.5±27.71
SevMea	73	0	73.33	19.45±18.11

SymSevSca	73	0	21	6.45±5.92

X±SS: Mean± Standart Deviation, ICIQTot: ICIQ-SF total point, 100mlSwal: 100 ml water swalloving speed, EAT-10: Eating Assessment Tool-10 GeHea: General Health InIm:Incontinence impact RoLim: Role Limitation PhyLim:Physical limitation SoLim: Social limitation Emo: Emotions SleEne: Sleep/energy SevMea: Severity Measurements SymSevSca: Symptom Severity Scale

The UI status of the participants is shown in Figure 1. Based on the ICIQ-SF question 'When does urine leak?', 16.44% of participants (n=12) reported no urine leakage. Among the 83.56% of participants (n=61) who experienced some form of incontinence, the most frequently reported circumstances were: leakage when you are asleep (20.55%, n=15), leakage when coughing or sneezing (17.81%, n=13), leakage during physical activity (15.07%, n=11), leakage when you have finishing urination and are dressed (9.59%, n=7), leakage before reaching the toilet (8.22%, n=6), leaks all the time (6.85%, n=5), and leakage for no obvious reason (5.48%, n=4).

Figure 1 is here

Figure 1. Distribution of urinary incontinence patterns among nursing home residents (n=73). Values represent percentage of the total sample and number of participants.

Multiple linear regression analysis was conducted to examine the association between UI and the independent variables: presence/absence of presbyphagia, age, and gender. In the multiple linear regression analyses with UI and I-QOL as dependent variables, a high correlation was observed between gender and handgrip strength (r = -.959), and the VIF values were 12.55 and 12.54, respectively. Since these values exceeded the recommended threshold of 5, gender and handgrip strength could not be included simultaneously in any of the regression models (20).To ensure model reliability, handgrip strength was removed from the analyses.

The multiple linear regression analysis revealed that the model including presbyphagia, age, and gender was significantly associated with UI [F(3,69)= 5.33, p=0.002], explaining 15.3% of the variance. Accordingly, presence/absence of presbyphagia was

significantly associated with UI (β= 0.39, t(69)=3.53, p=0.001, pr^2=0.15), while age (β= -0.02, t(69)=-0.18, p=0.85, pr^2=0.0004) and gender (β= 0.2, t(69)=1.85, p=0.06, pr^2=0.04) showed no significant association (Table 2).

Table 2. Regression coefficients of age, gender, presbyphagia group in ICIQ-SF

**Independent Variable**	**B [95% CI]**	**SE**	**β**	**p**	**Adjusted R**
Constant	-.73 [-9.62, 8.16]	4.45	–	.87	
Presbyphagia	3.28 [1.42, 5.13]	.92	.39	.001*	.15
Age	-.01 [-.12, .1]	.05	-.02	.85	
Gender	1.65 [-.12, 3.44]	.89	.2	.06	

R = 0.43; R^2 = 0.18; F = 5.33 p = 0.002 Durbin-Watson = 1.66*, B: unstandardized regression coefficient β: standardized regression coefficient, SE standard error; (Reference categories: Presbyphagia = no; Gender = male)

Multivariable binary logistic regression analysis revealed that participants with presbyphagia had 7.47 times higher odds of UI (OR = 7.47, 95% CI: 2.24–24.94, p = 0.001) compared to those without presbyphagia, after adjusting for age and gender. Male gender was also significantly associated with higher UI odds (OR = 5.16, 95% CI: 1.53–17.42, p = 0.008), while age was not significant (OR = 0.96, 95% CI: 0.90–1.04, p = 0.329). The model was statistically significant (χ^2 = 21.53, df = 3, p < 0.001) and correctly classified 71.2% of cases (Table 3).

Table 3. Multivariabl Binary Logistic Regression Analysis for Factors Associated with UI Presence

Variable	B (SE)	Wald	p	OR	95% CI for OR	
					Lower	Upper
Presbyphagia	2.01 (0.62)	10.69	.001	7.47	2.24	24.94
Age	-0.04 (0.04)	0.95	.329	0.96	0.9	1.04
Gender	1.64 (0.62)	6.97	.008	5.16	1.53	17.42
Constant	1.59 (2.94)	0.29	.588	4.92		

B = unstandardized coefficient. SE = standard error. OR = odds ratio. CI = confidence interval. ref: non-presbyphagia, (ref: female); Model fit: $\chi^2(3)$ = 21.53, p < .001; Cox & Snell R^2 = .26; Nagelkerke R^2 = .35; correctly classified cases = 71.2%

Multiple linear regression analysis revealed that the model including presbyphagia, age, and gender was significantly associated with all I-QOL sub-dimension, (except for the sleep/energy) explaining between 9.6% and 20% of the variance. Accordingly, the presence/absence of presbyphagia was positively and significantly associated with several I-

QOL sub-dimensions: general health (β = 0.33, t(69) = 2.87, p = 0.005, pr^2 = 0.10), incontinence impact (β = 0.41, t(69) = 3.71, p=0.004, pr^2 = 0.16), role limitation (β = 0.43, t(69) = 3.98, p=0.0001, pr^2 = 0.18), physical limitation (β = 0.39, t(69) = 3.62, p= 0.001, pr^2 = 0.16), social limitation (β = 0.33, t(69) = 2.91, p = 0.0048, pr^2 = 0.10), emotion (β = 0.41, t(69) = 3.70, p=0. 0004, pr^2 = 0.16), and symptom severity scale (β = 0.34, t(69) = 2.67, p = 0.004, pr^2 = 0.09). No significant associations were found between age or gender and any KHQ sub-dimension (p>0.005). (Table 4). There was no autocorrelation among the independent variables (1.5 < Durbin-Watson value > 2.5). There was no multicollinearity issue since handgrip strength was not included in the analyses based on VIF values.

Table 4. Regression coefficients of age, gender, presbyphagia group in KHQ sub-dimension

Dependent Variable	Independent Variable	B [95% CI]	SE	β	*p*	AdjR	F
General health	Constant	11.30 [-38.79, 61.39]	25.11	---	.65	.096	3.55
	Presbyphagia	15.01 [4.58, 25.45]	5.23	.33	.005*		
	Age	-.19 [-.84, .44]	.32	-.07	.54		
	Gender	8.43 [-1.59, 18.45]	5.02	.18	.09		
Incontinence impact	Constant	-27.41 [-76,52, 24.69]	26.12	---	.29	.17	6.1
	Presbyphagia	20.23 [9.37, 31.09]	5.44	.41	.0004*		
	Age	.05 [-.61, .72]	.33	.01	.87		
	Gender	9.49[-.48, 20.38]	5.22	.2	.06		
Role Limitation	Constant	-18.86 [-47.07, 9.33]	14.13	---	.18	.18	6.44
	Presbyphagia	11.74[5.86, 17.61]	2.94	.43	.0001*		
	Age	.06 [-.30, .42]	.18	.03	.74		
	Gender	3.81[-1.83, 9.45]	2.83	.14	.18		
Physical limitation	Constant	-35.59 [-76.68, 5.49]	20.59	---	.08	.19	6.69
	Presbyphagia	15.55 [6,99, 24.11]	4.29	.39	.001*		
	Age	.22[-.3, .75]	.26	.09	.4		
	Gender	8.23 [.01, 16.46]	4.12	.21	.05		
Social limitation	Constant	-8.32 [-42.69, 26.04]	17.23	---	.63	.12	4.43
	Presbyphagia	10.45 [3.29, 17.61]	3.59	.33	.0048*		
	Age	-.06 [-.50, .39]	.22	-.03	.80		
	Gender	7.75 [.87, 14.63]	3.45	.25	.03		
Personel Relationship	Constant	12.08 [1.65, 22.51]	5.23	---	.02	.02	1.47
	Presbyphagia	.68 [-1.50, 2.85]	1.09	.08	.54		
	Age	-.14 [-.27, .003]	.07	-.25	.04		
	Gender	-.40 [-2.49, 1.69]	1.05	-.04	.70		
Emotions	Constant	-21.43 [-74.60, 31.74]	26.65	---	.42	.19	6.76
	Presbyphagia	20.56 [9.48, 31.63]	5.55	.41	.0004*		
	Age	-.13 [-.81, .56]	.34	-.04	.72		
	Gender	14.11[3.47, 24.75]	5.34	.28	.01		

Sleep/energy	Constant	36.42 [-28.56, 101.4]	32.57	---	.27	-.005	.88
	Presbyphagia	10.28 [-3.25, 23.82]	6.79	.19	.13		
	Age	-.33 [-1.17, .51]	.42	-.09	.44		
	Gender	3.44 [-9.57, 16.45]	6.52	.06	.6		
Severity Measurements	Constant	17.94 [-22.25, 58.14]	20.15	---	.38	.1	3.65
	Presbyphagia	11.24 [2.86, 19.61]	4.20	.31	.009		
	Age	-.35 [-.87, .17]	.26	-.16	.18		
	Gender	7.95 [-.09, 16.00]	4.03	.22	.053		
Symptom Severity Scale	Constant	-4.49 [-17.59, 8.60]	6.56	---	.5	.1	3.83
	Presbyphagia	4.02 [1.29, 6.74]	1.37	.34	.0045*		
	Age	.03 [-.14, .20]	.08	.05	.69		
	Gender	1.71 [-.91, 4.33]	1.31	.15	.20		

B: unstandardized regression coefficient β: standardized regression coefficient, SE standard error; (Reference categories: Presbyphagia = no; Gender = male)

The pearson correlation analysis revealed a negative and significant correlation between age and KHQ personel sub-dimensions (r=-.22 p=.05). Point biceral correlation analysis revealed a positive and significant relationship between Presbyphagia and ICIQ-SF total score (r=.38 p=.001) and KHQ sub-dimensions [general health (r=.3 p=.008), incontinence impact (r=.41 p<.001), role limitation (r=.44 p<.001), physical limitation (r=.41 p<.001), social limitation (r=.31 p=.006), emotions (r=.38 p=.001), severity measurements (r=.26 p=.02), symptom severity scale (r=.34 p=.003)]. In addition, a positive and significant correlation was found between gender and KHQ sub-dimensions [Social limitation (r=.23 p=.04), Emotions (r=.26 p=.02)] (Table 5).

Table 5. The correlation between dependent and independent variables

		Age[a]	Presbyphagia[b]	Gender[b]
ICIQTot	r	.08	.38	.18
	p	.46	.001*	.11
GeHea	r	.02	.3	.17
	p	.86	.008*	.14
InIm	r	.13	.41	.19
	p	.27	.000*	.1
RoLim	r	.15	.44	.12
	p	.19	.000*	.27
PhyLim	r	.2	.41	.2
	p	.08	.000*	.08
SoLim	r	.06	.31	.23
	p	.59	.006*	.04⸸
Personel	r	-.22	.14	-.05
	p	.05⸸	.9	.64
Emo	r	.07	.38	.26
	p	.54	.001*	.02⸸

SleEne	r	-.04	.15	.05
	p	.7	.17	.65
SevMea	r	-.06	.26	.2
	p	.56	.02⸸	.08
SymSevSca	r	.13	.34	.13
	p	.24	.003*	.25

r Pearson correlation, p test statistic value, [a] Pearson correlation analysis, [b] Point-biserial correlation analysis, *p<.001 ⸸p<0.05

4. DİSCUSSİON

This study investigated the association between presbyphagia and UI and I-QOL in a distinct population of healthy elderly residents within Turkish nursing home. The presence of presbyphagia was found to be independent associated with UI and poor I-QOL in healthy elderly people living in nursing homes. Notably, presbyphagia was independently associated with 7.47-fold higher odds of UI. Age was not found to have any association with UI and I-QOL. There was no significant association between the gender variable and I-QOL. In contrast, binary logistic regression analysis examining the categorical presence of UI revealed that male gender was independently associated with a 5.16-fold higher odds of UI compared to females. This study reveals an independent association between presbyphagia and UI, providing a basis for hypotheses on the interplay between oral frailty and urinary health.

Yoneyama et al reported a strong association of presbyphagia with UI independent of age (12). The results of our study are consistent with Yoneyama et al. This association may be related to anatomical and functional similarity of autonomic nerve fibers in the gastrointestinal and genitourinary systems, particularly regulatory role of the autonomic nervous system on detrusor muscle, sphincter control (21) and oropharyngeal muscles (22). Another factor that may contribute to the association between UI and presbyphagia may be inadequate fluid intake due to SD. Elderly people with presbyphagia may consume less fluid to avoid complications such as coughing or choking. This may have potentially impaired the optimal function of the bladder and contributed to mucosal dryness and increased urine density (23).

De Jesus Fidyk et al. reported a strong association with UI in nursing home residents who had difficulty in swallowing solid food using questionnaires (7). Given the early stage uncertainty of presbyphagia and its tendency to be overlooked clinically (3); we considered that the De Jesus Fidyk study may have included participants with severe swallowing

dysfunction. In our study of individuals diagnosed with early stage presbyphagia but without significant symptoms, we observed a similar association to that found in De Jesus Fidyk et al. Thus, our findings suggest that the association between presbyphagia and UI might begin at an earlier stage than previously thought. De Jesus Fidyk et al. reported that the odds of UI was 13.8 times higher in participants with dysphagia who stayed in a nursing home for two years or longer, while this rate was 7.47 times odds of UI in our study (7). This difference may be related to the fact that long-term stay in a nursing home is associated with higher odds of UI (24). The wide confidence interval observed in the multivariable binary logistic regression reflects the relatively small sample size and the exploratory nature of this study; however, the lower bound (2.24) remains substantially above 1, supporting the robustness and statistical significance of the association between presbyphagia and UI risk.

Previous studies have reported that swallowing and oral health disorders are associated with poor health-related quality of life (HrQOL). Oral and dental health problems related to presbyphagia have been linked to increased psychological burden, avoidance of social and physical activities, weakening of social relationships and a decreased overall life satisfaction, especially in the elderly in nursing homes. These studies have reported that impaired oral health triggers systemic diseases and dramatically reduces quality of life by disrupting nutrition (25, 26). Although there are studies in the literature investigating the association of SD with HrQOL, studies evaluating the I-QOL relationship are limited. In our study, the presence of presbyphagia was found to be associated with all sub-dimensions of I-QOL (except personel relationships, severity measurements and sleep sub-dimension). The participants' personal relationships may have been positively influenced by living with their peers in the nursing home and their sleep quality might be related to staying in single rooms.. The association between presbyphagia and I-QOL provides a hypothesis-generating foundation for future research investigating potential shared mechanisms underlying these

conditions. Furthermore, given the association between presbyphagia and UI, we suggest that future studies investigating I-QOL consider the UI confounder.

UI is generally considered more common and associated with female gender and older age, and most studies focus on this population (27, 28). Contrary to the traditional view, no relationship was found between age and UI in our study, and the association with gender revealed a notable pattern. The study showed that gender had no significant association with the severity of urinary incontinence, but male gender was strongly and independently associated with the presence of urinary incontinence (OR = 5.16). This suggests that although UI prevalence is higher among male nursing home residents, its clinical severity is comparable between genders once incontinence develops. This finding aligns with previous studies reporting that gender differences in UI diminish with aging in institutionalized populations (14, 29). Yavuz and Etiler reported that the male/female UI ratio was 1.4 in Turkish nursing home residents aged 65–74 years (29). Similarly, Offermans et al. found no significant relationship between gender and UI in nursing home residents (8), and Aggazzotti et al. noted that UI risk increases in both genders at older ages (30). A possible explanation for the higher UI prevalence in males may be urinary retention (31), but common risks shared by both gender—such as physical limitations (5), multiple drug use and pain (32) may have masked the gender association with UI severity.

The finding that male gender was associated with approximately 5-fold higher odds of UI in our study is consistent with Matsumoto et al. (33), who reported an independent association between chronic periodontal disease and lower urinary tract symptoms, particularly in males. Our study extends this finding from a relatively simple oral condition—periodontal disease—to a more complex oral dysfunction: presbyphagia. Both studies identify male sex as an important factor associated with UI, contrasting with the traditional focus on female gender in the general population. While Matsumoto et al. attributed this male-specific

association to systemic inflammation-induced muscle weakness, our analysis—which included handgrip strength as a covariate— provides preliminary evidence that the presbyphagia-UI link is independent of generalized muscle weakness. These findings suggest that oral health in its broad spectrum-from periodontal status to swallowing function- may be relevant to UI, and that presbyphagia warrants greater clinical attention as a potential indicator of UI risk in especially older males adults.

As with gender, age also did not emerge as a significant factor associated with UI severity in our study. Although Kessler et al. reported an association between age and UI risk in individuals over 75 years of age (34), no significant association between age and UI was found in the participants of our study, whose mean age was 76.5 years. A possible explanation for this finding may be that environmental factors such as regular toilet access and staff support in a nursing home setting may mitigate the adverse effects of advanced age by alleviating UI symptoms. Our findings indicate that the relationship between presbyphagia and UI and I-QOL should be evaluated in conjunction with other factors.

This study has several limitations. First, the cross-sectional design precludes the establishment of causal relationships. Second, the data were collected from non-profit nursing homes in a single city in Turkey, which may limit the generalizability of the findings. Third, self-reported data are susceptible to recall and response biases, as both presbyphagia and UI outcomes were assessed using symptom-based questionnaires. Fourth, medical records pertaining to swallowing complications were inconsistent. Fifthly, hydration status was not assessed in this study. Sixth, although we attempted to control for sarcopenia by including handgrip strength as a proxy measure, high multicollinearity between handgrip strength and gender (VIF > 5) necessitated its exclusion from the final regression models; therefore, residual confounding related to generalized muscle weakness cannot be completely ruled out. Seventh, the inability to interview all residents with cognitive impairments represents an

additional limitation. Lastly, although data on UI subtypes were collected, the small number of participants. Precluded subtype-specific statistical analyses, limiting our ability to examine potential associations between presbyphagia and different UI types.

5. CONCLUSIONS

This study provides novel evidence that presbyphagia is independently associated with UI and poorer I-QOL among functionally independent elderly nursing home residents. Individuals with presbyphagia had 7.47-fold higher odds of UI compared to those without presbyphagia. The association, which persisted after accounting for age, handgrip strength and gender, challenges the conventional focus on these traditional risk factors in institutional settings and suggests that oral frailty may offer a different perspective on pelvic health in the elderly. Regarding gender-related findings, while male gender was associated with approximately 5.16 -fold higher odds of UI presence, no significant gender differences were observed in UI severity, nor was gender significantly associated with I-QOL. This suggests that once incontinence develops, its clinical course is similar in both genders. Additionally, age was not significantly associated with UI presence, UI severity, or I-QOL.

However, several limitations should be acknowledged, including the modest sample size,, single-centre design, inability to perform UI subtype-specific analyses due to low urgency UI prevalence, lack of hydration status measurement, and exclusion of handgrip strength due to multicollinearity with gender. Additionally, both presbyphagia and UI outcomes were assessed using symptom-based questionnaires, which introduces the possibility that the observed associations may partially reflect overall symptom burden or response bias rather than a specific physiological link. Despite these limitations, it should not be overlooked that our study is exploratory and hypothesis-generating in nature. Accordingly, our findings highlight the need for future research with larger, more diverse cohorts incorporating comprehensive assessments of potential confounders including hydration status, sarcopenia measures, and UI subtypes to better understand the relationship between presbyphagia and UI. This study contributes to the understanding of the complex interaction

between presbyphagia and UI and provides a hypothesis-generating foundation for future research in this area.

Data availability statement: https://data.mendeley.com/preview/s9z78b585n?a=3cb7ca47-c4fc-46f3-bf99-d5098fc7f55d

Authors' Contribution: The author carried out all tasks alone.

Funding and Support Information: The author received no specific funding for this work.

Acknowledgements: We would like to thank Ayşegül Yıldız for her proofreading of the manuscript and for facilitating institutional communication.

Conflict of Interest Statement: The author declares that there is no conflict of interest.

6. REFERANCE

1. Parisius KG, Wartewig E, Schoonmade LJ, Aarab G, Gobbens R, Lobbezoo F. Oral frailty dissected and conceptualized: A scoping review. Arch Gerontol Geriatr. 2022;100:104653.
2. Cai J, Gong Z, Zhang Y, Wang H, Niu C, Dai Y. The prevalence of presbyphagia in older adults: a systematic review and meta-analysis. Wien Klin Wochenschr. 2024;136(17):497–506.
3. Feng H-Y, Zhang P-P, Wang X-W. Presbyphagia: Dysphagia in the elderly. World journal of clinical cases. 2023;11(11):2363.
4. Nogueira D, Reis E. Swallowing disorders in nursing home residents: how can the problem be explained? Clin Interv Aging. 2013;8(null):221–7.
5. Farrés-Godayol P, Jerez-Roig J, Minobes-Molina E, Yildirim M, Molas-Tuneu M, Escribà-Salvans A, et al. Urinary incontinence and its association with physical and psycho-cognitive factors: a cross-sectional study in older people living in nursing homes. Int J Environ Res Public Health. 2022;19(3):1500.
6. Huang P, Luo K, Wang C, Guo D, Wang S, Jiang Y, et al. Urinary incontinence is associated with increased all-cause mortality in older nursing home residents: a meta-analysis. J Nurs Scholarsh. 2021;53(5):561–7.
7. de Jesus Fidyk S, de Oliveira MLC, Ramos LB, Roriz AKC, Ribeiro SML, Ferreira LdNM, et al. Path analysis of urinary incontinence in older adults living in nursing homes. Sci Rep. 2024;27(10(1)):5553.
8. Offermans MP, Du Moulin MF, Hamers JP, Dassen T, Halfens RJ. Prevalence of urinary incontinence and associated risk factors in nursing home residents: a systematic review. Neurourol Urodyn. 2009;28(4):288–94.
9. Azzolino D, Damanti S, Bertagnoli L, Lucchi T, Cesari M. Sarcopenia and swallowing disorders in older people. Aging Clin Exp Res. 2019;31(6):799–805.
10. Kakehi S, Wakabayashi H, Isono E, Takemura R, Sato Y, Otsuka Y, et al. Association between sarcopenia and urinary dysfunction in patients with dysphagia. Arch Gerontol Geriatr. 2024;127:105577.
11. Widjanantie SC, Attamimy V. Functional Breathing and Approaches in Pulmonary Rehabilitation: A Literature Review. Indonesian Journal of Physical Medicine and Rehabilitation. 2024;13(01):74–86.
12. Yoneyama F, Okamoto T, Tamura Y, Ishii N, Togashi K, Soma O, et al. Association between oral frailty and lower urinary tract symptoms among middle-aged and older adults in community-dwelling individuals: a cross-sectional study. Int Urol Nephrol. 2024;56(6):1803–10.
13. Gürsoy Z, Seçginli S. An investigation of healthy aging among older adults in nursing homes: A cross-sectional study. Geriatric Nursing. 2025;65:103466.
14. Jerez-Roig J, Farrés-Godayol P, Yildirim M, Escribà-Salvans A, Moreno-Martin P, Goutan-Roura E, et al. Prevalence of urinary incontinence and associated factors in nursing homes: a multicentre cross-sectional study. BMC Geriatr. 2024;24(1):169.
15. Wu M-C, Chang Y-C, Wang T-G, Lin L-C. Evaluating swallowing dysfunction using a 100-ml water swallowing test. Dysphagia. 2004;19:43–7.
16. Demir N, Serel Arslan S, İnal Ö, Karaduman AA. Reliability and Validity of the Turkish Eating Assessment Tool (T-EAT-10). Dysphagia. 2016;31(5):644–9.
17. Çetinel B, Ozkan B, Can G. The validation study of ICIQ-SF Turkish version. Turk J Urol. 2004;30(3):332–8.

18. Kaya S, Akbayrak T, Toprak Çelenay Ş, Dolgun A, Ekici G, Beksaç S. Reliability and validity of the Turkish King's Health Questionnaire in women with urinary incontinence. International Urogynecology Journal. 2015;26(12):1853–9.
19. Kim JH. Multicollinearity and misleading statistical results. kja. 2019;72(6):558–69.
20. Craney TA, Surles JG. Model-Dependent Variance Inflation Factor Cutoff Values. Quality Engineering. 2002;14(3):391–403.
21. Conde-Santos G, Padilla-Fernández B. Targeting the Autonomic Nervous System for Treatment of Urinary Incontinence. European Urology Focus. 2025.
22. Souza LR, Oliveira MV, Basile JR, Souza LN, Souza AC, Haikal DS, et al., editors. Anatomical and physiopathological aspects of oral cavity and oropharynx components related to oropharyngeal dysphagia. Seminars in dysphagia; 2015: IntechOpen.
23. Ney DM, Weiss JM, Kind AJ, Robbins J. Senescent swallowing: impact, strategies, and interventions. Nutr Clin Pract. 2009;24(3):395–413.
24. Saga S, Vinsnes AG, Mørkved S, Norton C, Seim A. What characteristics predispose to continence in nursing home residents?: A population-based cross-sectional study. Neurourol Urodyn. 2015;34(4):362–7.
25. Janto M, Iurcov R, Daina CM, Neculoiu DC, Venter AC, Badau D, et al. Oral Health among Elderly, Impact on Life Quality, Access of Elderly Patients to Oral Health Services and Methods to Improve Oral Health: A Narrative Review. Journal of Personalized Medicine. 2022;12(3):372.
26. Porter J, Ntouva A, Read A, Murdoch M, Ola D, Tsakos G. The impact of oral health on the quality of life of nursing home residents. Health and Quality of Life Outcomes. 2015;13(1):102.
27. Savas S, Saka B, Akın S, Tasci I, Tasar PT, Tufan A, et al. The prevalence and risk factors for urinary incontinence among inpatients, a multicenter study from Turkey. Arch Gerontol Geriatr. 2020;90:104122.
28. Abufaraj M, Xu T, Cao C, Siyam A, Isleem U, Massad A, et al. Prevalence and trends in urinary incontinence among women in the United States, 2005–2018. Am J Obstet Gynecol. 2021;225(2):166. e1–. e12.
29. Yavuz M, Etiler N. Addressing urinary incontinence by gender: a nationwide population-based study in Turkiye. BMC Urol. 2023;23(1):205.
30. Aggazzotti G, Pesce F, Grassi D, Fantuzzi G, Righi E, De Vita D, et al. Prevalence of urinary incontinence among institutionalized patients: a cross-sectional epidemiologic study in a midsized city in northern Italy. Urology. 2000;56(2):245–9.
31. Condon M, Mannion E, Molloy DW, O'Caoimh R. Urinary and faecal incontinence: point prevalence and predictors in a university hospital. Int J Environ Res Public Health. 2019;16(2):194.
32. Kim YH, Kim SH, Kim T, Ma R. Health-related Physical Fitness, Blood Pressure, and Body Mass Index among People with Intellectual Disability, Visual Impairment, and Hearing Impairment. Exerc Sci. 2024;33(1):93–105.
33. Matsumoto S, Matsuda M, Takekawa M, Okada M, Hashizume K, Wada N, et al. Association between Chronic Periodontal Disease and Lower Urinary Tract Symptoms in Both Sexes. Low Urin Tract Symptoms. 2015;7(1):17–21.
34. Kessler M, Facchini LA, Soares MU, Nunes BP, França SM, Thumé E. Prevalence of urinary incontinence among the elderly and relationship with physical and mental health indicators. Revista Brasileira de Geriatria e Gerontologia. 2018;21(04):397–407.

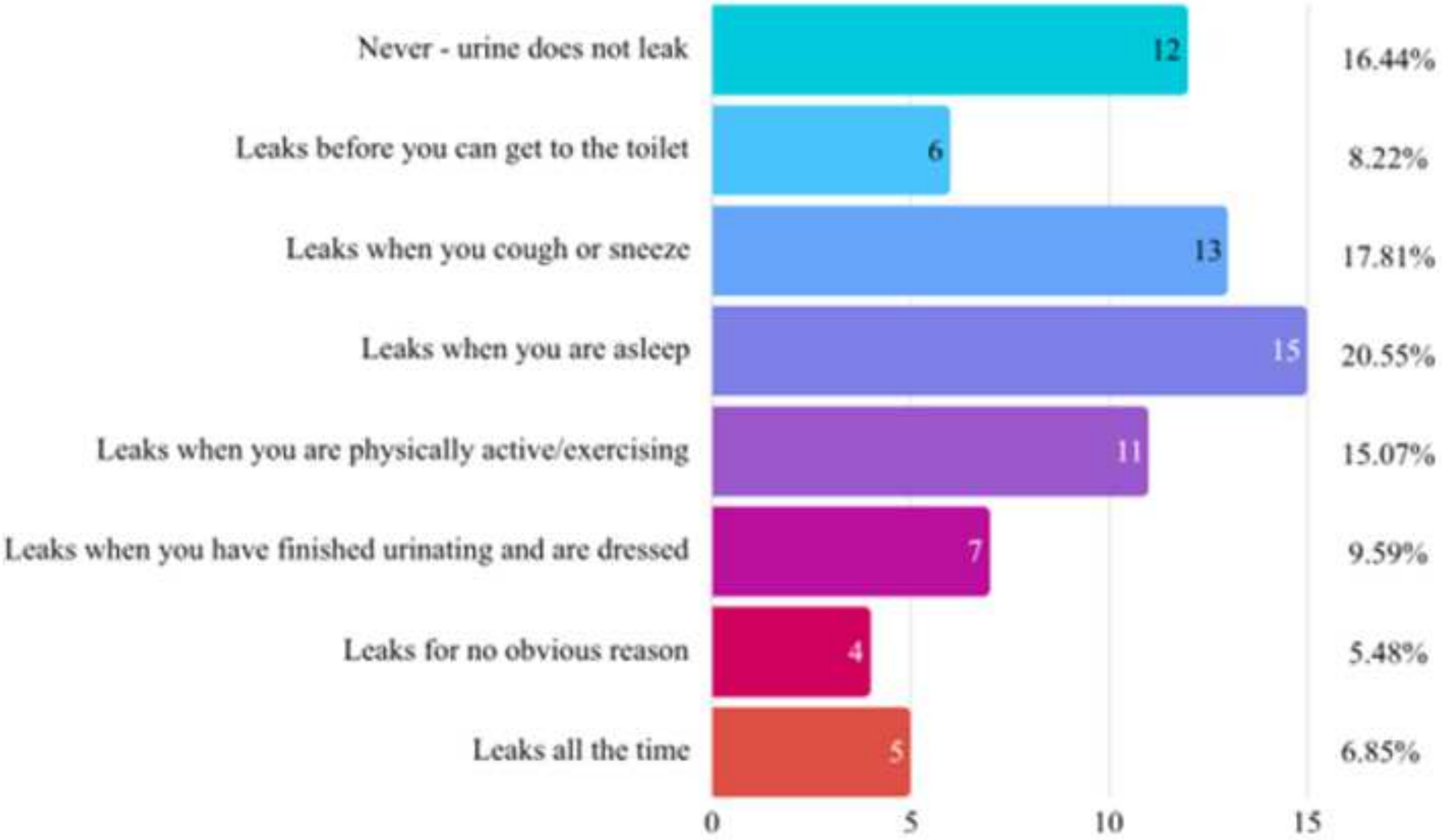
Never - urine does not leak
Leaks before you can get to the toilet
Leaks when you cough or sneeze
Leaks when you are asleep
Leaks when you are physically active/exercising
Leaks when you have finished urinating and are dressed
Leaks for no obvious reason
Leaks all the time
12
6
13
15
11
7
4
5
0
5
10
15
16.44%
8.22%
17.81%
20.55%
15.07%
9.59%
5.48%
6.85%

Click here to access/download
Supporting Information
renamed_38332.pdf

Click here to access/download
Supporting Information
renamed_899dd.pdf

The Association of Presbyphagia with Urinary Incontinence and Incontinence-Related Quality of Life in Nursing Home Residents: A Cross-Sectional Study

Presbyphagia in the elderly

Ziya YILDIZ[1*]

[1*] PhD, Department of Physical Therapy and Rehabilitation, Uluborlu Vocational School, Isparta University of Applied Sciences, 32050, Isparta, TURKEY
Telephone: +90 507 507 7573

***Correspondence:** ziyayildiz@isparta.edu.tr

ORCID: https://orcid.org/0000-0001-6961-8202

Abstract

Purpose: The association between presbyphagia (age-related swallowing decline) and urinary incontinence (UI) remains poorly understood. This study therefore investigated the relationship between presbyphagia and both UI and incontinence-specific quality of life (I-QOL) in a distinct population of functionally independent healthy elderly in nursing home.

Method: The presence of presbyphagia was determined in 73 participants in the nursing home using the 100ml water swallowing test (100mlSwal) and the Eating Assessment Tool-10 (EAT-10) tests. UI was determined using the International Consultation on Incontinence Questionnaire Short (ICIQ-SF) and I-QOL was determined using the King's Health Questionnaire (KHQ). The presence of presbyphagia, age and female gender confounding factors were analysed by multiple regression analysis. Multiple linear and binary logistic regression analyses were performed with presbyphagia, age, gender, and handgrip strength. Binary logistic regression was used to calculate odds ratios for UI presence.

Results: Presbyphagia was significantly associated with UI (p=.001). Individuals with presbyphagia had 7.47-fold higher odds of UI. Presbyphagia was also significantly associated with multiple I-QOL sub-domains, including incontinence impact, role limitation, physical limitation, and emotions (all p<.001). Male gender was associated with higher UI presence

(OR=5.16), but not with UI severity. Age showed no significant associations with UI or I-QOL.~~The association between presbyphagia and UI (p=.001) was found in elderly individuals in nursing homes. In addition, presbyphagia and I-QOL was significantly associated with general health (p=.005), incontinence effect (p<.001), role limitation (p<.001), physical limitation (p=.001), social limitation (p=.005), emotions (p<.001), severity measures (p=.01), symptom severity scale (p<.001) sub-dimensions.~~

Conclusion: ~~This study revealed significant associations between presbyphagia and both UI and I-QOL dimensions in functionally independent nursing home residents, while showing no such associations with age or gender. These findings highlight presbyphagia as a relevant factor in the UI context. Future UI research should include assessments of swallowing function, and clinical evaluations might consider examining both urinary and swallowing functions concurrently in elderly care.~~Presbyphagia is independently associated with UI presence and poorer I-QOL in nursing home residents. Male gender was associated with higher UI prevalence but similar severity and I-QOL to females. These findings suggest an independent association between presbyphagia and UI, thereby contributing to the formulation of hypotheses aimed at understanding the complex interactions between oral frailty and the urinary system. Further research is needed to elucidate underlying mechanisms and explore the potential clinical implications of this association.

Formatted: Indent: First line: 0", Line spacing: Double

Keywords: Swallowing disorder, Oral ~~fractality~~frailty, Presbyphagia, Urinary incontinence, Incontinence-related quality of life

***The clinical trials number of the study is NCT06827561.

1. INTRODUCTİON

UI and swallowing disorder (SD) are common problems in healthy elderly individuals living in nursing homes. ~~In the elderly, reduced intraoral pressure, oropharyngeal dysfunction and anatomical changes in the head/neck region can lead to a SD called presbyphagia or oral frailty~~One such SD is presbyphagia, defined as the structural, physiological, and innervational alterations in the swallowing process that occur as a result of healthy aging. These age-related changes—including progressive brain atrophy, neurological deterioration, and loss of muscle mass and strength in the oropharyngeal region—can lead to clinical symptoms such as choking, coughing, pharyngeal residue, aspiration pneumonia and impaired swallowing rate (1, 2). ~~Presbyphagia leads to clinical symptoms such as choking, coughing, pharyngeal residue, aspiration pneumonia and impaired swallowing rate.~~ Presbyphagia is an overlooked health problem in the elderly because it is perceived as part of the normal aging process and develops secretly (3). ~~Presbyphagia~~ It is the primary contributing factor to aspiration pneumonia in 50-75% of nursing home residents and is more common in this population compared to community-dwelling elderly (4).

UI, which is considered a health priority by the World Health Organization, is another problem that emerges with aging (5). A multicenter study reported that the prevalence of UI in nursing homes was approximately 70% (6). UI, which is caused by weakness in the nerve and muscle structure (7), is an important cause of poor quality of life in the elderly in nursing homes (5). UI causes weaknesses such as decreased interpersonal relationships, loss of career and psychological well-being, limitations in the ability to exercise, and impaired sexual life (8).

The studies focusing UI and SD in the elderly have been associated with sarcopenia through different mechanisms (8, 9). However, this relationship between UI and sarcopenia in elderly patients with SD was not found (10). Theories such as the “soda pop model”, which

explains the anatomical relationship between the pelvic floor, diaphragm and pharynx/larynx, attempt to theorize the complex interaction between UI and swallowing function (11). However, the literature explaining the relationship and mechanism between presbyphagia and UI and I-QOL remains limited. Although there is a study associating presbyphagia with lower urinary tract symptoms in middle-aged/elderly Japanese community-dwelling populations (12), research focusing on elderly individuals in nursing homes, particularly in middle-income countries like Turkey where institutional residency often reflects socioeconomic status rather than health status, is notably absent. This population is particularly relevant given recent findings reporting a healthy ageing rate of 38.5% in Turkish nursing homes (13), indicating a substantial subgroup of healthy older adults in these settings.

In addition, effect modifiers and confounders associated with UI in nursing homes have not been adequately researched due to reasons such as facility characteristics, staffing issues, reimbursement and cultural attitudes (8). Although UI is known to be hidden behind multiple diseases, weakness or other geriatric syndromes, (14) its relationship with presbyphagia has not been fully established. Therefore, this study aimed to investigate the relationship between presbyphagia and UI and I-QOL in this novel population of healthy elderly nursing home residents. Within this context, we tested the hypothesis that UI and I-QOL would be significantly associated with presbyphagia.

2. METHOD

2.1 Study design and setting

This cross-sectional observational study was conducted between December 23-30, 2024 using clinical tests and a face-to-face interview survey instrument. Participants have provided written informed consent. The study included healthy elderly individuals aged 65-95 years at Gardenya Nursing Home and Hacı Ahmet Şençopur Nursing Home in Isparta, Turkey. The study was approved by the Research Ethics Committee of Isparta University of

Applied Sciences on November 4, 2024 (CASE: E-96714346-050.04-139027, evaluation number: 203/5). The study was conducted in accordance with Declaration of Helsinki for research involving humans and the STROBE guidelines for conducting observational studies. Mini mental state examination was used to determine the mental status of the participants. After demographic characteristics of the volunteers were recorded, 100mlSwal and EAT-10 scale were used to determine SD, International Consultation on Incontinence Questionnaire Short Form (ICIQ-SF) was used to determine UI, and King's Health Questionnaire (KHQ) was used to determine the I-QOL.

2.2 Participants and Sample Size

The required sample size was calculated a priori using G*Power 3.1 software. For a multiple linear regression analysis with a significance level (α) of 0.05, a statistical power (1-β) of 0.95, and a high effect size ($f^2 = 0.35$), a minimum of 40 participants was required. The study initially assessed 298 elderly individuals from the nursing home for eligibility.

The inclusion criteria were: (1) being a resident of the nursing home for at least one year, (2) a Mini-Mental State Examination (MMSE) score of 24 or above, indicating no significant cognitive impairment, (3) the absence of advanced hearing or visual problems that would hinder communication, and (4) provision of voluntary informed consent.

The exclusion criteria were designed to ensure a sample of functionally independent older adults without conditions that could directly confound the study's outcomes. Individuals were excluded if they met any of the following criteria: (1) dependency in any of the Basic Activities of Daily Living as assessed by the Katz ADL Index (ındex point<6), (2) a history of falls within the past 6 months or requiring assistance for ambulation, (3) a known history of neurological or psychiatric diseases (e.g., stroke, Parkinson's disease, dementia, or major depressive disorder defined by a score >10 on the 15-item Geriatric Depression Scale), (4) a history of diseases or surgeries with a known direct impact on swallowing or UI mechanisms,

(5) regular use of medications that could significantly affect lower urinary tract function or cognitive status (e.g., diuretics, anticholinergics, antipsychotics), (6) an acute or uncontrolled systemic illness (e.g., uncontrolled hypertension, decompensated heart failure), (7) a diagnosed urinary tract infection within the past 4 weeks.

Based on these criteria, 225 individuals were excluded. The final study sample consisted of 73 participants. A post-hoc power analysis was conducted using the observed effect size ($f^2 = 0.17$) from the study data. This analysis confirmed that the study achieved a high statistical power of 97% at a 0.05 significance level, thus validating the robustness of the findings despite the observed effect size being lower than the initially anticipated high effect size used for the a priori calculation.

2.3 Measurements

EAT-10 scale and 100mlSwal test were used in the evaluation of presbyphagia. The 100mlSwal test was measured by sitting the participants on a chair with armrests and asking them to drink water quickly and not to hold it in the mouth. The time until the thyroid cartilage returned to its resting position was recorded for participants who had completely finished the water. The swallowing rate (ml/s) was calculated for all participants by dividing the amount of water drunk by the time recorded. Abnormal swallowing rate was considered to be below 10 ml/s. (15). If the sum of the scores given to each item in the EAT-10 scale was 3 points or more, the risk of SD was accepted. Presbyphagia was considered to be present in healthy elderly individuals who scored 3 points or more on the EAT-10 scale (16) or who scored less than 10 ml/sec on the 100mlSwal test (15).

The ICIQ-SF is a scale assesses the frequency, severity in UI. A Likert scale is used to assess the presence/absence and severity of symptoms. The overall score range is 0 to 21, with higher values indicating increased symptom severity. The cut-off score to distinguish between those with and without UI is 6 (17).

KHQ was used to calculate the change in I-QOL. In the first part, Role, Physical, and Social Limitations, Limitations in Personal Relationship, Emotional Problems, Sleep and Energy Disturbances are evaluated. The second part includes the Symptom Severity Scale, which assesses the presence and severity of urinary symptoms (18).

2.4. Statistical Analysis

The data obtained were analysed using version 26.0 of the SPSS programme. Missing data were identified and corrected during data collection and entry. The skewness and kurtosis values of the data were checked for the assumption of normality, and the values were found to range between − 2.0 and + 2.0. The analysis was performed with presbyphagia/non-presbyphagia group, age, handgrip strength and gender as independent variables and UI and I-QOL as dependent variables. Multiple linear regression analysis was performed to explain the relationship between the variables. The R-squared (R^2) statistic evaluates the extent to which the regression model explains the variation in the dependent variable. The unstandardized beta coefficient (B) indicates the magnitude and direction of the relationship between the independent variables and the dependent variable. Pearson correlation and point-biserial correlation analysis was performed to determine the relationship between variables. Bonferroni correction was applied selectively to balance the risks of type 1 and type 2 errors. For the 10 KHQ subdimension—a coherent set of related outcomes assessing incontinence-specific quality of life—the significance threshold was set at $p < 0.005$ (0.05/10). The ICIQ-SF analysis for UI presence was considered a separate, independent hypothesis and was not subjected to multiple comparisons correction, consistent with standard practice for distinct research questions ($p<0.05$)~~. The statistical significance in all analyses was determined according to the p<0.05 level.~~ Multivariable Binary logistic regression was used to assess the association between presbyphagia (0 = non-presbyphagia, 1 = presbyphagia) and UI presence (ICIQ-SF ≥6; 0 = no, 1 = yes), adjusting for age and gender. Handgrip strength was excluded

from the regression models due to high multicollinearity with gender (r = -.959; VIF = 12.55 and 12.54 for gender and handgrip strength, respectively), which exceeded the recommended threshold of 5 and could compromise model stability (19). Results are presented as adjusted odds ratios (OR) with 95% confidence intervals (CI). Finally, odds ratios (ORs) adjusted for relative risk were calculated.

2.5. Validity and Reliability

The questionnaires were administered by a single researcher. The internal consistency of the Turkish EAT-10 scale was found to be 0.90 Cronbach's alpha and the test repeatability was 0.91 Cronbach's alpha (16). The ICIQ-SF is a scale with a validity level of A. The reliability and validity of the Turkish version was calculated as Chronbach's alpha value of 0.71 (17). The internal consistency (Cronbach's alpha value ≥0.68) and test-retest reliability of the Turkish version of the KHQ were found to be high (18).

3. RESULT

A total of 73 white elderly, 35 males and 38 females, participated in the study. The study included 32 participants with presbyphagia (16 males, 16 females) and 41 participants without presbyphagia (19 males, 22 females). The mean age of the participants was 76.53± 8.05 years. The mean body mass index was 24.08±1.2 kg/m^2. The mean swallowing rate was 15.4±5.32 ml/sec. Total ICIQ-SF score was 5.68±4.13 and Symptom Severity Scale was 6.45±5.92. (Table 1). The 100mlSwa test rate was 15.4± 5.32 ml/sn (Table 1). There were 32 participants diagnosed with presbyphagia, including 4 participants with a 100mlSwa test rate below 10 ml/sec, 19 participants with an EAT-10 score of 3 and above, and 9 participants who failed both tests.

Formatted: Not Highlight

Formatted: Not Highlight

Table 1. General information of the participants

	n	Minimum	Maximum	X± SS
Age (year)	73	65	92	76.5±8.05
ICIQTot	73	0	18	5.68±4.13

100mlSwal (ml/sn)	73	6.7	27	15.4±5.32
EAT-10	73	0	7	2.31±1.61
Handgrip strength	73	15.7	32.1	23.14±5.72
GeHea	73	0	75	30.47±22.53
InIm	73	0	100	21±24.53
RoLim	73	0	66.67	8.44±13.35
PhyLim	73	0	83.33	16.43±19.54
SoLim	73	0	50	14.1±15.7
Emo	73	0	88.89	19.39±25.32
SleEne	73	0	83.33	31.5±27.71
SevMea	73	0	73.33	19.45±18.11
SymSevSca	73	0	21	6.45±5.92

X±SS: Mean± Standart Deviation, ICIQTot: ICIQ-SF total point, 100mlSwal: 100 ml water swalloving speed, EAT-10: Eating Assessment Tool-10 GeHea: General Health InIm:Incontinence impact RoLim: Role Limitation PhyLim:Physical limitation SoLim: Social limitation Emo: Emotions SleEne: Sleep/energy SevMea: Severity Measurements SymSevSca: Symptom Severity Scale

The UI status of the participants is shown in Figure 1. Based on the ICIQ-SF question 'When does urine leak?', 16.44% of participants (n=12) reported no urine leakage. Among the 83.56% of participants (n=61) who experienced some form of incontinence, the most frequently reported circumstances were: leakage when you are asleep (20.55%, n=15), leakage when coughing or sneezing (17.81%, n=13), leakage during physical activity (15.07%, n=11), leakage when you have finishing urination and are dressed (9.59%, n=7), leakage before reaching the toilet (8.22%, n=6), leaks all the time (6.85%, n=5), and leakage for no obvious reason (5.48%, n=4).

Figure 1 is here

Figure 1. ~~The situations in which UI occurred in the participants~~ Distribution of urinary incontinence patterns among nursing home residents (n=73). Values represent percentage of the total sample and number of participants.

Multiple linear regression analysis was conducted to examine the association between ~~was performed to predict the KHQ sub-dimensions and~~ UI and the independent variables: presence/absence of presbyphagia, age, and gender. ~~dependent variables using the independent variables of presence/absence of presbyphagia, age and gender.~~ In the multiple linear regression analyses with UI and I-QOL as dependent variables, a high correlation was

Formatted: Indent: First line: 0.49"

Formatted: Line spacing: Double

Formatted: Font: Not Bold

Formatted: Font: Not Bold

observed between gender and handgrip strength (r = -.959), and the VIF values were 12.55 and 12.54, respectively. Since these values exceeded the recommended threshold of 5, gender and handgrip strength could not be included simultaneously in any of the regression models (20).~~ ~~To ensure model reliability, handgrip strength was removed from the analyses.

The multiple linear regression analysis revealed that the model including presbyphagia, age, and gender was significantly associated with UI [F(3,69)= 5.33, p=0.002], explaining 15.3% of the variance. ~~As a result of the analysis, a significant regression model [F(3,69)= 5.33, p=0.002] was determined that 15.3% of the variance in the dependent variable was predicted by the independent variables. Accordingly, presence/absence of presbyphagia positively and significantly predicted UI (β= 0.39 t (69)=3.53, p=0.001 pr2=0.15). Age (β= -0.02 t (69)=-0.18, p=0.85 pr2=0.0004) and gender (β= 0.2 t (69)=1.85, p=0.06 pr^2=0.04) did not significantly predict UI (Table 2).~~ Accordingly, presence/absence of presbyphagia was significantly associated with UI (β= 0.39, t(69)=3.53, p=0.001, pr^2=0.15), while age (β= -0.02, t(69)=-0.18, p=0.85, pr^2=0.0004) and gender (β= 0.2, t(69)=1.85, p=0.06, pr^2=0.04) showed no significant association (Table 2). ~~In addition, participants were classified as having or not having UI based on the ICIQ-SF cut-off value and the odds ratio was calculated. Participants were classified as having/not having UI according to the ICIQ-SF cut-off value and an odds ratio of 4.61 was calculated.~~

Table 2. Regression coefficients of age, gender, presbyphagia group in ICIQ~~-~~SF

Independent Variable	B [95% CI]	SE	β	p	~~95% CI~~		Adjusted R
					~~Lower~~	~~Upper~~	
Constant	-.73 [-9.62, 8.16]	4.45	–	.87	~~-9.62~~	~~8.16~~	.15
Presbyphagia	3.28 [1.42, 5.13]	.92	.39	.001*	~~1.42~~	~~5.13~~	
Age	-.01 [-.12, .1]	.05	-.02	.85	~~-.12~~	~~.1~~	
Gender	1.65 [-.12, 3.44]	.89	.2	.06	~~-.12~~	~~3.44~~	

R = 0.43; R^2 = 0.18; F = 5.33 p = 0.002 Durbin-Watson = 1.66*~~ α significance level is taken as 0.05~~, B: unstandardized regression coefficient β: standardized regression coefficient, SE standard error; ~~95% CI, 95% confidence intervals, ~~(Reference categories: Presbyphagia = no; Gender = male~~Presbyphagia Group reference value= non-predisphajia Gender reference value=male~~)

Formatted Table

Multivariable binary logistic regression analysis revealed that participants with presbyphagia had 7.47 times higher odds of UI (OR = 7.47, 95% CI: 2.24–24.94, p = 0.001) compared to those without presbyphagia, after adjusting for age and gender. Male gender was also significantly associated with higher UI odds (OR = 5.16, 95% CI: 1.53–17.42, p = 0.008), while age was not significant (OR = 0.96, 95% CI: 0.90–1.04, p = 0.329). The model was statistically significant (χ^2 = 21.53, df = 3, p < 0.001) and correctly classified 71.2% of cases (Table 3).

Table 3. Multivariabl Binary Logistic Regression Analysis for Factors Associated with UI Presence

Variable	B (SE)	Wald	p	OR	95% CI for OR	
					Lower	Upper
Presbyphagia	2.01 (0.62)	10.69	.001	7.47	2.24	24.94
Age	-0.04 (0.04)	0.95	.329	0.96	0.9	1.04
Gender	1.64 (0.62)	6.97	.008	5.16	1.53	17.42
Constant	1.59 (2.94)	0.29	.588	4.92		

B = unstandardized coefficient. SE = standard error. OR = odds ratio. CI = confidence interval. ref: non-presbyphagia, (ref: female); Model fit: $\chi^2(3)$ = 21.53, p < .001; Cox & Snell R^2 = .26; Nagelkerke R^2 = .35; correctly classified cases = 71.2%

Multiple linear regression analysis revealed that the model including presbyphagia, age, and gender was significantly associated with all I-QOL sub-dimension, (except for the sleep/energy) explaining between 9.6% and 20% of the variance. ~~A significant regression model was found to explain between 9.6% and 20% of the variance in the dependent variable by the independent variables, except for the KHQ slepp/energy subscale. Accordingly, the presence/absence of presbyphagia was determined in terms of general health (β= 0.33 t (69)=2.87, p=0.005 pr2=0.1), incontinence impact (β= 0.41 t (69)=3. 71, p=0.0004 pr2=0.16), role limitation (β= 0.43 t (69)=3.98, p=0.0001 pr2=0.18), physical limitation (β= 0.39 t (69)=3.62, p=0.001 pr2=0. 16), social limitation (β= 0.33 t (69)=2.91, p=0.005 pr2=0.1), emotion (β= 0.4 t (69)=3.7, p=0.0004 pr2=0.16), severity measurements (β= 0.18 t (69)=1. 51, p=0.01 pr2=0.03), symptom severity scale (β= 0.31 t (69)=2.67, p=0.009 pr2=0.09) predicted positively and significantly.~~ Accordingly, the presence/absence of presbyphagia was

Formatted
Formatted
Formatted Table
Formatted
Formatted
Formatted
Formatted
Formatted
Formatted
Formatted
Formatted
Formatted
Formatted
Formatted
Formatted
Formatted
Formatted
Formatted
Formatted
Formatted
Formatted
Formatted
Formatted Table
Formatted
Formatted
Formatted
Formatted
Formatted
Formatted
Formatted
Formatted
Formatted
Formatted
Formatted
Formatted
Formatted
Formatted
Formatted
Formatted
Formatted
Formatted
Formatted
Formatted Table
Formatted
Formatted

positively and significantly associated with several I-QOL sub-dimensions: general health (β = 0.33, t(69) = 2.87, p = 0.005, pr² = 0.10), incontinence impact (β = 0.41, t(69) = 3.71, p=0.004, pr² = 0.16), role limitation (β = 0.43, t(69) = 3.98, p=0.0001, pr² = 0.18), physical limitation (β = 0.39, t(69) = 3.62, p= 0.001, pr² = 0.16), social limitation (β = 0.33, t(69) = 2.91, p = 0.0048, pr² = 0.10), emotion (β = 0.41, t(69) = 3.70, p=0. 0004, pr² = 0.16), and symptom severity scale (β = 0.34, t(69) = 2.67, p = 0.004, pr² = 0.09). No significant associations were found between age or gender and any KHQ sub-dimension (p>0.005). Age negatively and significantly predicted only personnel (β= -0.24 t (69)=-2.04, p=0.04 pr2=0.05). Gender positively and significantly predicted only social limitation (β= 0.24 t (69)=2.24, p=0.02 pr2=0.06), and emotion (β= 0.28 t (69)=2.64, p=0.01 pr2=0.09) (Table 5.4). There was no autocorrelation among the independent variables (1.5 < Durbin-Watson value > 2.5). There were no multiple connection problems according to Variance inflation factors (VIF) values (VIF < 10). There was no multicollinearity issue since handgrip strength was not included in the analyses based on VIF values.

Table 34. Regression coefficients of age, gender, presbyphagia group in KHQ sub-dimension

Dependent Variable	Independent Variable	B [95% CI]	SE	β	*p*	95% CI Lower	95% CI Upper	Adj R	F
General health	Constant	11.30 [-38.79, 61.39]	25.11	---	.65	-38.79	61.39	.096	3.55
	Presbyphagia	15.01 [4.58, 25.45]	5.23	.33	.005*	4.58	25.45		
	Age	-.19 [-.84, .44]	.32	-.07	.54	-.84	.44		
	Gender	8.43 [-1.59, 18.45]	5.02	.18	.09	-1.59	18.45		
Incontinence impact	Constant	-27.41 [-76,52, 24.69]	26.12	---	.29	-76,52	24.69	.17	6.1
	Presbyphagia	20.23 [9.37, 31.09]	5.44	.41	.0004*	9.37	31.09		
	Age	.05 [-.61, .72]	.33	.01	.87	-.61	.72		
	Gender	9.49[-.48, 20.38]	5.22	.2	.06	-.48	20.38		
Role Limitation	Constant	-18.86 [-47.07, 9.33]	14.13	---	.18	-47.07	9.33	.18	6.44
	Presbyphagia	11.74[5.86, 17.61]	2.94	.43	.0001*	5.86	17.61		
	Age	.06 [-.30, .42]	.18	.03	.74	-.30	.42		
	Gender	3.81[-1.83,	2.83	.14	.18	-1.83	9.45		

Formatted Table

Formatted: Font: Not Italic

Formatted: Font: Italic

Formatted Table

		9.45]							
Physical limitation	Constant	-35.59 [-76.68, 5.49]	20.59	---	.08	-76.68	5.49	.19	6.69
	Presbyphagia	15.55 [6,99, 24.11]	4.29	.39	.001*	6,992	24.11		
	Age	.22[-.3, .75]	.26	.09	.4	-.3	.75		
	Gender	8.23 [.01, 16.46]	4.12	.21	.05	.01	16.46		
Social limitation	Constant	-8.32 [-42.69, 26.04]	17.23	---	.63	-42.69	26.04	.12	4.43
	Presbyphagia	10.45 [3.29, 17.61]	3.59	.33	.0050048*	3.29	17.61		
	Age	-.06 [-.50, .39]	.22	-.03	.80	-.50	.39		
	Gender	7.75 [.87, 14.63]	3.45	.25	.03*	.87	14.63		
Personel Relationship	Constant	12.08 [1.65, 22.51]	5.23	---	.02	1.65	22.51	.02	1.47
	Presbyphagia	.68 [-1.50, 2.85]	1.09	.08	.54	-1.50	2.85		
	Age	-.14 [-.27, .003]	.07	-.25	.04*	-.27	.003		
	Gender	-.40 [-2.49, 1.69]	1.05	-.04	.70	-2.49	1.69		
Emotions	Constant	-21.43 [-74.60, 31.74]	26.65	---	.42	-74.60	31.74	.19	6.76
	Presbyphagia	20.56 [9.48, 31.63]	5.55	.41	.0004 *	9.48	31.63		
	Age	-.13 [-.81, .56]	.34	-.04	.72	-.81	.56		
	Gender	14.11[3.47, 24.75]	5.34	.28	.01*	3.47	24.75		
Sleep/energy	Constant	36.42 [-28.56, 101.4]	32.57	---	.27	-28.56	101.4	-.005	.88
	Presbyphagia	10.28 [-3.25, 23.82]	6.79	.19	.13	-3.25	23.82		
	Age	-.33 [-1.17, .51]	.42	-.09	.44	-1.17	.51		
	Gender	3.44 [-9.57, 16.45]	6.52	.06	.6	-9.57	16.45		
Severity Measurements	Constant	17.94 [-22.25, 58.14]	20.15	---	.38	-22.25	58.14	.1	3.65
	Presbyphagia	11.24 [2.86, 19.61]	4.20	.31	.01.009*	2.86	19.61		
	Age	-.35 [-.87, .17]	.26	-.16	.18	-.87	.17		
	Gender	7.95 [-.09, 16.00]	4.03	.22	.053	-.09	16.00		
Symptom Severity Scale	Constant	-4.49 [-17.59, 8.60]	6.56	---	.5	-17.59	8.60	.1	3.83
	Presbyphagia	4.02 [1.29, 6.74]	1.37	.34	.0045 *	1.29	6.74		
	Age	.03 [-.14, .20]	.08	.05	.69	-.14	.20		
	Gender	1.71 [-.91, 4.33]	1.31	.15	.20	-.91	4.33		

Formatted: Font color: Auto

Formatted: Font color: Auto

α significance level is taken as 0.05, B: unstandardized regression coefficient β: standardized regression coefficient, SE standard error; 95% CI, 95% confidence intervals, (Reference categories: Presbyphagia = no; Gender = malePresbyphagia Group reference value= non-predisphajia Gender reference value=male)

The pearson correlation analysis revealed a negative and significant correlation between age and KHQ personel sub-dimensions (r=-.22 p=.05). Point biceral correlation analysis revealed a positive and significant relationship between Presbyphagia and ICIQ-SF total score (r=.38 p=.001) and KHQ sub-dimensions [general health (r=.3 p=.008), incontinence impact (r=.41 p<.001), role limitation (r=.44 p<.001), physical limitation (r=.41 p<.001), social limitation (r=.31 p=.006), emotions (r=.38 p=.001), severity measurements (r=.26 p=.02), symptom severity scale (r=.34 p=.003)]. In addition, a positive and significant correlation was found between gender and KHQ sub-dimensions [Social limitation (r=.23 p=.04), Emotions (r=.26 p=.02)] (Table 54).

Formatted: Line spacing: Double

Table 45. The correlation between dependent and independent variables (n=73)

		Age[a]	Presbyphagia[b]	Gender[b]
ICIQTot	r	.08	.38	.18
	p	.46	.001*	.11
GeHea	r	.02	.3	.17
	p	.86	.008*	.14
InIm	r	.13	.41	.19
	p	.27	.000*	.1
RoLim	r	.15	.44	.12
	p	.19	.000*	.27
PhyLim	r	.2	.41	.2
	p	.08	.000*	.08
SoLim	r	.06	.31	.23
	p	.59	.006*	.04⸸
Personel	r	-.22	.14	-.05
	p	.05⸸	.9	.64
Emo	r	.07	.38	.26
	p	.54	.001*	.02⸸
SleEne	r	-.04	.15	.05
	p	.7	.17	.65
SevMea	r	-.06	.26	.2
	p	.56	.02⸸	.08
SymSevSca	r	.13	.34	.13
	p	.24	.003*	.25

r Pearson correlation, p test statistic value, [a] Pearson correlation analysis, [b] Point-biserial correlation analysis, *p<.001 ⸸p<0.05

4. DİSCUSSİON

This study investigated the association between presbyphagia and UI and I-QOL in a distinct population of healthy elderly residents within ~~a~~ Turkish nursing home. The presence of presbyphagia was found to be independent associated with UI and poor I-QOL in healthy elderly people living in nursing homes. Notably, presbyphagia was independently associated with 7.47-fold higher odds of UI. ~~Aging~~ Age was not found to have any association with UI and I-QOL. ~~(except for the personal relationships subscale).~~ There was no significant association between the gender variable and I-QOL. ~~There was no association between being female and UI, whereas a significant association was found in the social limitation and emotion sub-dimensions of I-QOL.~~ In contrast, binary logistic regression analysis examining the categorical presence of UI revealed that male gender was independently associated with a 5.16 fold higher odds of UI compared to females. ~~This study provides a new insight into the complex interactions between oral frailty and the urinary system by demonstrating an independent association between presbyphagia and UI.~~ This study reveals an independent association between presbyphagia and UI, providing a basis for hypotheses on the interplay between oral frailty and urinary health.

Yoneyama et al reported a strong association of presbyphagia with UI independent of age (12). The results of our study are consistent with Yoneyama et al. This association may be ~~explained~~ related to ~~by the~~ anatomical and functional similarity of autonomic nerve ~~fibres~~ fibers in the gastrointestinal and genitourinary systems~~. –~~, particularly ~~It may be due to the~~ regulatory role of the autonomic nervous system on detrusor muscle, sphincter control (21) and oropharyngeal muscles (22). Another factor that may contribute to the ~~reason for the~~ association between UI and presbyphagia may be inadequate fluid intake due to SD. Elderly people with presbyphagia may consume less fluid to avoid complications such as coughing or

Formatted: Space After: 6 pt

choking. This may have potentially impaired the optimal function of the bladder and contributed to mucosal dryness and increased urine density (23).

De Jesus Fidyk et al. reported a strong association with UI in nursing home residents who had difficulty in swallowing solid food using questionnaires (7). Given the early stage uncertainty of presbyphagia and its tendency to be overlooked clinically (3); we considered that the De Jesus Fidyk study may have included participants ~~consisted of participants~~ with severe swallowing dysfunction. In our study of individuals diagnosed with early-stage presbyphagia but without significant symptoms, we observed a similar association to that found in De Jesus Fidyk et al. ~~In our study, early stage presbyphagia with no obvious symptoms was identified by evaluating with quantitative scales.~~ Thus, our findings ~~indicate~~ suggest that the association between presbyphagia and UI might begin at an earlier stage than previously thought. De Jesus Fidyk et al. reported that the ~~risk~~ odds of UI was 13.8 times higher in participants with dysphagia who stayed in a nursing home for two years or longer, while this rate was ~~4.61~~7.47 times ~~higher~~odds of UI in our study (7). This difference may be related to the ~~explained by the~~ fact that long-term stay in a nursing home is associated with higher odds~~increases the risk~~ of UI (24). The wide confidence interval observed in the multivariable binary logistic regression reflects the relatively small sample size and the exploratory nature of this study; however, the lower bound (2.24) remains substantially above 1, supporting the robustness and statistical significance of the association between presbyphagia and UI risk.

~~Matsumoto et al. demonstrated an independent association between chronic periodontal disease and UI severity in middle aged individuals (25).~~ .~~This finding suggests that even a relatively simple oral condition, like periodontal disease, may be linked to UI. In our study, the association between presbyphagia, a more complex oral dysfunction, and UI suggests that presbyphagia should be taken more seriously in the clinic.~~

Formatted: Space After: 6 pt

Formatted: Font: Not Bold

Previous studies have ~~It has been~~ reported that swallowing and oral health disorders are associated with~~lead to~~ poor health-related quality of life (HrQOL). Oral and dental health problems related ~~leading~~ to presbyphagia have been linked to ~~cause an~~ increased ~~in~~ psychological burden, avoidance of social and physical activities, weakening of social relationships and a decreased ~~in~~ overall life satisfaction, especially in the elderly in nursing homes. These studies have reported that impaired oral health triggers systemic diseases and dramatically reduces quality of life by disrupting nutrition (26, 27). Although there are studies in the literature investigating the association of SD with HrQOL, studies evaluating the I-QOL relationship are limited. In our study, the presence of presbyphagia was found to be associated with all sub-dimensions of I-QOL (except personel relationships, severity measurements and sleep sub-dimension). The participants' personal relationships may have been positively influenced by living ~~were not affected because they lived~~ with their peers in the nursing home and their sleep quality might be related to staying in single rooms.~~may not have deteriorated because they stayed in single rooms~~. The association between presbyphagia and I-QOL provides a hypothesis-generating foundation for future research investigating potential shared mechanisms underlying these conditions. ~~warrants investigation into potential combined intervention approaches.~~ Furthermore, given the association between presbyphagia and UI, we suggest that future studies investigating I-QOL consider the UI confounder.

Formatted: Line spacing: Double

~~UI is generally considered more common and associated with the female gender and older age, and most studies focus on this population~~UI is generally considered more common and associated with female gender and older age, and most studies focus on this population (28, 29). Contrary to the traditional view~~literature~~, no relationship was found between age and ~~gender and~~ UI in our study,and the association with gender revealed a notable pattern.~~.~~ The study showed that gender had no significant association with the severity of urinary incontinence, but male gender was strongly and independently associated with the presence of

urinary incontinence (OR = 5.16). ~~Furthermore, there are studies that support our results and report that gender and age are not associated with UI~~ This suggests that although UI prevalence is higher among male nursing home residents, its clinical severity is comparable between genders once incontinence develops. This finding aligns with previous studies reporting that gender differences in UI diminish with aging in institutionalized populations (14, 30). ~~Yavuz and Etiler reported that the difference in UI between genders decreased with ageing in nursing home elderly in Turkey. The ratio of male/female UI in 65-74 years of age was found to be 1.4~~ (30)~~.~~ Yavuz and Etiler reported that the male/female UI ratio was 1.4 in Turkish nursing home residents aged 65–74 years (30). Similarly, Offermans et al. found no significant relationship between gender and UI in nursing home residents (8), and Aggazzotti et al. noted that UI risk increases in both genders at older ages (31). ~~Similarly, it has been reported that there is no significant relationship between UI and gender in nursing home residents~~ (8) ~~and that the risk increases in both genders at older ages~~ (31). A possible explanation for the higher UI prevalence in males may be urinary retention (32), but common risks shared by both gender—such as physical limitations (5), multiple drug use and pain (33) may have masked the gender association with UI severity . ~~The reason for the lack of a association between gender and UI in our study may be the high prevalence of urinary retention in elderly men~~ (32)~~. Common risks associated with both genders, such as physical limitations (5), polypharmacy and pain~~ (34) ~~may have masked the gender difference in our study.~~

The finding that male gender was associated with approximately 5-fold higher odds of UI in our study is consistent with Matsumoto et al.(25), who reported an independent association between chronic periodontal disease and lower urinary tract symptoms, particularly in males. The our study extends this finding from a relatively simple oral condition—periodontal disease—to a more complex oral dysfunction: presbyphagia. Both

Formatted: Space After: 6 pt

Formatted: Not Highlight

studies identify male sex as an important factor associated with UI, contrasting with the traditional focus on female gender in the general population. While Matsumoto et al. attributed this male-specific association to systemic inflammation-induced muscle weakness, our analysis—which included handgrip strength as a covariate— provides preliminary evidence that the presbyphagia-UI link is independent of generalized muscle weakness. These findings suggest that oral health in its broad spectrum-from periodontal status to swallowing function- may be relevant to UI, and that presbyphagia warrants greater clinical attention as a potential indicator of UI risk in especially older males adults.

As with gender, age also did not emerge as a significant factor associated with UI severity in our study. Although Kessler et al. reported an association between age and UI risk in individuals over 75 years of age (35), no significant association between age and UI was found in the participants of our study, whose mean age was 76.5 years. A possible explanation for this finding may be that environmental factors such as regular toilet access and staff support in a nursing home setting may mitigate the adverse effects of advanced age by alleviating UI symptoms. Our findings indicate that the relationship between presbyphagia and UI and I-QOL should be evaluated in conjunction with other factors.

This study has several limitations. First, the cross-sectional design precludes the establishment of causal relationships. Second, the data were collected from non-profit nursing homes in a single city in Turkey, which may limit the generalizability of the findings. Third, self-reported data are susceptible to recall and response biases, as both presbyphagia and UI outcomes were assessed using symptom-based questionnaires.~~Third, self-reported data are susceptible to recall and response biases~~. Fourth, medical records pertaining to swallowing complications were inconsistent. Fifthly, hydration status was not assessed in this study. ~~Lastly, the inability to interview all residents with cognitive impairments represents an additional limitation.~~ Sixth, although we attempted to control for sarcopenia by including

Formatted: Indent: First line: 0.49"

handgrip strength as a proxy measure, high multicollinearity between handgrip strength and gender (VIF > 5) necessitated its exclusion from the final regression models; therefore, residual confounding related to generalized muscle weakness cannot be completely ruled out. Seventh, the inability to interview all residents with cognitive impairments represents an additional limitation. Lastly, although data on UI subtypes were collected, the small number of participants. Precluded subtype-specific statistical analyses, limiting our ability to examine potential associations between presbyphagia and different UI types.

Formatted: Indent: First line: 0"

5. CONCLUSIONS

This study provides novel evidence that presbyphagia is independently associated with UI and poorer I-QOL among functionally independent elderly nursing home residents. Individuals with presbyphagia had 7.47-fold higher odds of UI compared to those without presbyphagia. The ~~strength of this~~ association, which persisted after accounting for age, handgrip strength ~~-~~and gender, challenges the conventional focus on these traditional risk factors in institutional settings and suggests ~~shifts~~ that oral frailty may offer a different perspective on~~attention towards oral frailty as a potential key determinant of~~ pelvic health in the elderly. Regarding gender-related findings, while male gender was associated with approximately 5.16 -fold higher odds of UI presence, no significant gender differences were observed in UI severity, nor was gender significantly associated with I-QOL. This suggests that once incontinence develops, its clinical course is similar in both genders. Additionally, age was not significantly associated with UI presence, UI severity, or I-QOL.

Formatted: Line spacing: Double

However, several limitations should be acknowledged, including the modest sample size,, single-center design, inability to perform UI subtype-specific analyses due to low urgency UI prevalence, lack of hydration status measurement, and exclusion of handgrip strength due to multicollinearity with gender. Additionally, both presbyphagia and UI outcomes were assessed using symptom-based questionnaires, which introduces the possibility that the observed associations may partially reflect overall symptom burden or response bias rather than a specific physiological link. Despite these limitations, it should not be overlooked that our study is exploratory and hypothesis-generating in nature. Accordingly, our findings highlight the need for future research with larger, more diverse cohorts incorporating comprehensive assessments of potential confounders including hydration status, sarcopenia measures, and UI subtypes to better understand the relationship between presbyphagia and UI. This study contributes to the understanding of the complex interaction

between presbyphagia and UI and provides a hypothesis-generating foundation for future research in this area.

The primary implication of our findings is that the clinical management of UI in nursing homes may be incomplete without an assessment of swallowing function. We propose that presbyphagia screening be integrated into comprehensive geriatric assessments as a standard practice. This could facilitate earlier identification of at-risk individuals for whom targeted interventions might be beneficial.

Formatted: Indent: First line: 0.49"

Future research should prioritize longitudinal studies to elucidate the temporal relationship and directionality between presbyphagia and UI. Furthermore, interventional trials are warranted to determine whether rehabilitative strategies aimed at improving swallowing function can concurrently ameliorate urinary symptoms and enhance quality of life in this population

Formatted: Indent: First line: 0"

Data availability statement: The raw data presented in the article can be shared openly upon request.https://data.mendeley.com/preview/s9z78b585n?a=3cb7ca47-c4fc-46f3-bf99-d5098fc7f55d

Authors' Contribution: The author carried out all tasks alone.

Funding and Support Information: The author received no specific funding for this work.

Acknowledgements: We would like to thank Ayşegül Yıldız for her proofreading of the manuscript and for facilitating institutional communication.

Conflict of Interest Statement: The author declares that there is no conflict of interest.

6. REFERANCE

1. Parisius KG, Wartewig E, Schoonmade LJ, Aarab G, Gobbens R, Lobbezoo F. Oral frailty dissected and conceptualized: A scoping review. Arch Gerontol Geriatr. 2022;100:104653.
2. Cai J, Gong Z, Zhang Y, Wang H, Niu C, Dai Y. The prevalence of presbyphagia in older adults: a systematic review and meta-analysis. Wien Klin Wochenschr. 2024;136(17):497–506.
3. Feng H-Y, Zhang P-P, Wang X-W. Presbyphagia: Dysphagia in the elderly. World journal of clinical cases. 2023;11(11):2363.
4. Nogueira D, Reis E. Swallowing disorders in nursing home residents: how can the problem be explained? Clin Interv Aging. 2013;8(null):221–7.
5. Farrés-Godayol P, Jerez-Roig J, Minobes-Molina E, Yildirim M, Molas-Tuneu M, Escribà-Salvans A, et al. Urinary incontinence and its association with physical and psycho-cognitive factors: a cross-sectional study in older people living in nursing homes. Int J Environ Res Public Health. 2022;19(3):1500.
6. Huang P, Luo K, Wang C, Guo D, Wang S, Jiang Y, et al. Urinary incontinence is associated with increased all-cause mortality in older nursing home residents: a meta-analysis. J Nurs Scholarsh. 2021;53(5):561–7.
7. de Jesus Fidyk S, de Oliveira MLC, Ramos LB, Roriz AKC, Ribeiro SML, Ferreira LdNM, et al. Path analysis of urinary incontinence in older adults living in nursing homes. Sci Rep. 2024;27(10(1)):5553.
8. Offermans MP, Du Moulin MF, Hamers JP, Dassen T, Halfens RJ. Prevalence of urinary incontinence and associated risk factors in nursing home residents: a systematic review. Neurourol Urodyn. 2009;28(4):288–94.
9. Azzolino D, Damanti S, Bertagnoli L, Lucchi T, Cesari M. Sarcopenia and swallowing disorders in older people. Aging Clin Exp Res. 2019;31(6):799–805.
10. Kakehi S, Wakabayashi H, Isono E, Takemura R, Sato Y, Otsuka Y, et al. Association between sarcopenia and urinary dysfunction in patients with dysphagia. Arch Gerontol Geriatr. 2024;127:105577.
11. Widjanantie SC, Attamimy V. Functional Breathing and Approaches in Pulmonary Rehabilitation: A Literature Review. Indonesian Journal of Physical Medicine and Rehabilitation. 2024;13(01):74–86.
12. Yoneyama F, Okamoto T, Tamura Y, Ishii N, Togashi K, Soma O, et al. Association between oral frailty and lower urinary tract symptoms among middle-aged and older adults in community-dwelling individuals: a cross-sectional study. Int Urol Nephrol. 2024;56(6):1803–10.
13. Gürsoy Z, Seçginli S. An investigation of healthy aging among older adults in nursing homes: A cross-sectional study. Geriatric Nursing. 2025;65:103466.
14. Jerez-Roig J, Farrés-Godayol P, Yildirim M, Escribà-Salvans A, Moreno-Martin P, Goutan-Roura E, et al. Prevalence of urinary incontinence and associated factors in nursing homes: a multicentre cross-sectional study. BMC Geriatr. 2024;24(1):169.
15. Wu M-C, Chang Y-C, Wang T-G, Lin L-C. Evaluating swallowing dysfunction using a 100-ml water swallowing test. Dysphagia. 2004;19:43–7.
16. Demir N, Serel Arslan S, İnal Ö, Karaduman AA. Reliability and Validity of the Turkish Eating Assessment Tool (T-EAT-10). Dysphagia. 2016;31(5):644–9.
17. Çetinel B, Ozkan B, Can G. The validation study of ICIQ-SF Turkish version. Turk J Urol. 2004;30(3):332–8.

18. Kaya S, Akbayrak T, Toprak Çelenay Ş, Dolgun A, Ekici G, Beksaç S. Reliability and validity of the Turkish King's Health Questionnaire in women with urinary incontinence. International Urogynecology Journal. 2015;26(12):1853–9.
19. Kim JH. Multicollinearity and misleading statistical results. kja. 2019;72(6):558–69.
20. Craney TA, Surles JG. Model-Dependent Variance Inflation Factor Cutoff Values. Quality Engineering. 2002;14(3):391–403.
21. Conde-Santos G, Padilla-Fernández B. Targeting the Autonomic Nervous System for Treatment of Urinary Incontinence. European Urology Focus. 2025.
22. Souza LR, Oliveira MV, Basile JR, Souza LN, Souza AC, Haikal DS, et al., editors. Anatomical and physiopathological aspects of oral cavity and oropharynx components related to oropharyngeal dysphagia. Seminars in dysphagia; 2015: IntechOpen.
23. Ney DM, Weiss JM, Kind AJ, Robbins J. Senescent swallowing: impact, strategies, and interventions. Nutr Clin Pract. 2009;24(3):395–413.
24. Saga S, Vinsnes AG, Mørkved S, Norton C, Seim A. What characteristics predispose to continence in nursing home residents?: A population-based cross-sectional study. Neurourol Urodyn. 2015;34(4):362–7.
25. Matsumoto S, Matsuda M, Takekawa M, Okada M, Hashizume K, Wada N, et al. Association between Chronic Periodontal Disease and Lower Urinary Tract Symptoms in Both Sexes. Low Urin Tract Symptoms. 2015;7(1):17–21.
26. Janto M, Iurcov R, Daina CM, Neculoiu DC, Venter AC, Badau D, et al. Oral Health among Elderly, Impact on Life Quality, Access of Elderly Patients to Oral Health Services and Methods to Improve Oral Health: A Narrative Review. Journal of Personalized Medicine. 2022;12(3):372.
27. Porter J, Ntouva A, Read A, Murdoch M, Ola D, Tsakos G. The impact of oral health on the quality of life of nursing home residents. Health and Quality of Life Outcomes. 2015;13(1):102.
28. Savas S, Saka B, Akın S, Tasci I, Tasar PT, Tufan A, et al. The prevalence and risk factors for urinary incontinence among inpatients, a multicenter study from Turkey. Arch Gerontol Geriatr. 2020;90:104122.
29. Abufaraj M, Xu T, Cao C, Siyam A, Isleem U, Massad A, et al. Prevalence and trends in urinary incontinence among women in the United States, 2005–2018. Am J Obstet Gynecol. 2021;225(2):166. e1–. e12.
30. Yavuz M, Etiler N. Addressing urinary incontinence by gender: a nationwide population-based study in Turkiye. BMC Urol. 2023;23(1):205.
31. Aggazzotti G, Pesce F, Grassi D, Fantuzzi G, Righi E, De Vita D, et al. Prevalence of urinary incontinence among institutionalized patients: a cross-sectional epidemiologic study in a midsized city in northern Italy. Urology. 2000;56(2):245–9.
32. Condon M, Mannion E, Molloy DW, O'Caoimh R. Urinary and faecal incontinence: point prevalence and predictors in a university hospital. Int J Environ Res Public Health. 2019;16(2):194.
33. Kim YH, Kim SH, Kim T, Ma R. Health-related Physical Fitness, Blood Pressure, and Body Mass Index among People with Intellectual Disability, Visual Impairment, and Hearing Impairment. Exerc Sci. 2024;33(1):93–105.
34. Kim KJ, Shin J, Choi J, Park JM, Park HK, Lee J, et al. Association of Geriatric Syndromes with Urinary Incontinence according to Sex and Urinary-Incontinence-Related Quality of Life in Older Inpatients: A Cross-Sectional Study of an Acute Care Hospital. Korean J Fam Med. 2019;40(4):235–40.
35. Kessler M, Facchini LA, Soares MU, Nunes BP, França SM, Thumé E. Prevalence of urinary incontinence among the elderly and relationship with physical and mental health indicators. Revista Brasileira de Geriatria e Gerontologia. 2018;21(04):397–407.

Response to Reviewer #1

Reviewer #1: "…However, there is a central point that deserves greater attention: presbyphagia and urinary incontinence frequently coexist because both emerge from the same pathophysiological context. This shared set of determinants creates a scenario in which any observed association between the two conditions is likely explained by confounding factors, especially sarcopenia. Since this important confounder was not analysed, even statistical adjustments cannot fully address this limitation, which compromises the interpretation of the results. Therefore, I recommend restructuring the text so that the findings related to the dependent variables—presbyphagia and urinary incontinence—are presented in relation to quality-of-life measures independently, avoiding causal inferences between presbyphagia and incontinence."

Answer: Thank you for your insightful comment regarding the shared pathophysiological background of presbyphagia and urinary incontinence, and the potential confounding role of sarcopenia. You have raised a very important point that we had not initially considered. In response to your valuable feedback, we recognized the need to control for sarcopenia in our analysis. Since handgrip strength measurements are routinely collected in our institution every three months and recorded in the institutional database, we obtained the values corresponding to the study period and attempted to incorporate them into our regression model as a proxy measure for sarcopenia. However, during the diagnostic process for multicollinearity, we encountered a critical statistical issue: a very high correlation between handgrip strength and gender (r = -.959) (Figure 1), resulting in Variance Inflation Factor (VIF) values of 12.55 and 12.54 (Figure 2), respectively. Since VIF values significantly exceeded the recommended threshold of 5 (Craney and Surles 2002), including both variables would have compromised the reliability and validity of our model. Consequently, despite our attempt to address your concern by obtaining and testing handgrip strength data, we had to exclude it from the final analysis due to these statistical limitations.

As noted by O'Brien (2007), when VIF values exceed recommended thresholds, researchers commonly address multicollinearity by removing one of the correlated variables, using ridge regression, or combining variables into a single index. Similarly, Kim (2019) emphasizes that excluding multicollinear predictors is an appropriate approach to obtain stable and reliable regression models. Following these established methodological recommendations, we chose to exclude handgrip strength from our analyses, as its high correlation with gender (r = -.959; VIF > 5) made simultaneous inclusion of both variables statistically problematic. This decision ensured the consistency and reliability of our regression models. As you recommended, we have now explicitly stated in the **Limitations section** that, although we attempted to include handgrip strength, sarcopenia could not be adequately controlled for as a potential confounder. Due to these changes and to avoid causal inferences, we have restructured the text.

Correlations

		ICIQTotal	Presbyphagia	Age	Gender	handgrip
Pearson Correlation	ICIQTotal	1,000	,384	,087	,187	-,201
	Presbyphagia	,384	1,000	,255	-,036	,015
	Age	,087	,255	1,000	,033	-,036
	Gender	,187	-,036	,033	1,000	-,959
	handgrip	-,201	,015	-,036	-,959	1,000
Sig. (1-tailed)	ICIQTotal	.	,000	,232	,057	,044
	Presbyphagia	,000	.	,015	,380	,452
	Age	,232	,015	.	,390	,380
	Gender	,057	,380	,390	.	,000
	handgrip	,044	,452	,380	,000	.
N	ICIQTotal	73	73	73	73	73
	Presbyphagia	73	73	73	73	73
	Age	73	73	73	73	73
	Gender	73	73	73	73	73
	handgrip	73	73	73	73	73

Figure 1. Correlations (handgrip vs gender)

Coefficients[a]

Model		Unstandardized Coefficients		Standardized Coefficients	t	Sig.	95,0% Confidence Interval for B		Correlations			Collinearity Statistics	
		B	Std. Error	Beta			Lower Bound	Upper Bound	Zero-order	Partial	Part	Tolerance	VIF
1	(Constant)	4,274	12,041		,355	,724	-19,753	28,302					
	Presbyphagia	3,252	,937	,393	3,472	,001	1,383	5,121	,384	,388	,379	,928	1,077
	Age	-,011	,058	-,021	-,185	,854	-,126	,105	,087	-,022	-,020	,933	1,072
	Gender	,294	3,176	,036	,093	,927	-6,045	6,632	,187	,011	,010	,080	12,559
	handgrip	-,125	,279	-,173	-,448	,655	-,682	,432	-,201	-,054	-,049	,080	12,542

a. Dependent Variable: ICIQTotal

Figure 2. VIF value

Reference:

Craney, T. A., & Surles, J. G. (2002). Model-Dependent Variance Inflation Factor Cutoff Values. *Quality Engineering*, *14*(3), 391–403. https://doi.org/10.1081/QEN-120001878

O'brien, R.M. A Caution Regarding Rules of Thumb for Variance Inflation Factors. *Qual Quant* **41**, 673–690 (2007). https://doi.org/10.1007/s11135-006-9018-6

Kim, J. H. (2019). Multicollinearity and misleading statistical results. *Korean journal of anesthesiology*, *72*(6), 558-569.

Response to Reviewer #2

Reviewer #2: “The abstract is slightly long and statistically dense. Abstract could be made little simpler..”

Answer: Thank you for your valuable feedback. We have revised the abstract to be more concise and less statistically dense. We believe this version is simpler and more accessible to readers.

Reviewer #2: “This is a cross-sectional study, but at several places the wording sounds like presbyphagia leads to urinary incontinence. Please be careful with language-Say “associated with”, not “predicts”, “affects”, or “should be screened as standard practice”. The conclusions and clinical suggestions should be more cautious and framed as hypothesis-generating, not practice-changing.”

Answer: a) Thank you for your insightful comment regarding the language used in our manuscript. You are absolutely right that in a cross-sectional study, terminology should reflect associations rather than causal relationships. Some of these wording issues arose during the translation process from our native language to English. We have now carefully revised the manuscript accordingly. For example:

Original: "Multiple linear analysis was performed to predict the UI dependent variables using the independent variable of presence/absence of presbyphagia, age and gender."

Revised: "Multiple linear regression analysis was conducted to examine the association between UI and the independent variables: presence/absence of presbyphagia, age, and gender."

b) In response to your comment, we have carefully revised the conclusion and discussion section accordingly. The overall tone of the conclusion now reflects a more cautious and hypothesis-generating perspective, highlighting the need for future longitudinal and interventional studies to confirm our findings.

Reviewer #2: Definition of presbyphagia needs clearer justification

Answer: We have substantially revised the introduction to provide a clearer, more comprehensive definition and justification of presbyphagia. The revised text now:

- Provides a precise, evidence-based definition of presbyphagia (structural, physiological, and innervational alterations)
- Specifies the underlying mechanisms (brain atrophy, neurological deterioration, muscle loss)
- Maintains connection to clinical symptoms with appropriate citations

We believe this revision addresses the reviewer's concern by clearly justifying what presbyphagia is and how it develops.

Reviewer #2: statistical analysis: The logistic regression model used to calculate the odds ratio is not clearly described Confidence intervals for the odds ratio are missing. Clearly state which analysis was used for the odds ratio. Report adjusted OR with 95% CI. Be consistent in how UI is analysed and interpreted

Answer: We thank the reviewers for their valuable comments regarding the odds ratio calculation and presentation. We have carefully addressed these points as follows:

- Removal of unclear odds ratio: The previous version of the manuscript included a statement: *'Participants were classified as having/not having UI according to the ICIQ-SF cut-off value and an odds ratio of 4.61 was calculated.'* We agree with the reviewers that this statement was insufficiently detailed, lacking information about the statistical method used, confidence intervals, and adjustment for confounders. This unclear statement has been removed entirely from the revised manuscript.
- Proper logistic regression analysis: In its place, we have now conducted and reported a binary logistic regression analysis following standard methodological guidelines. In the Statistical Analysis section, we clearly describe:
  Variable coding: presbyphagia (0 = non-presbyphagia, 1 = presbyphagia), UI presence (ICIQ-SF ≥6; 0 = no UI, 1 = UI present)
  Covariates: age and gender
  Exclusion of handgrip strength due to high multicollinearity with gender (VIF > 5, r = -0.96)
- Complete reporting of results: In the Results section, we now present the adjusted odds ratio with its 95% confidence interval: OR = 7.47 (95% CI: 2.24–24.94, p = 0.001). A detailed table (Table 3) following simplified APA format now includes B coefficients, standard errors, Wald statistics, p-values, odds ratios, and confidence intervals for all variables. Model fit statistics ($\chi^2 = 21.53$, df = 3, $p < 0.001$; Cox & Snell $R^2 = 0.26$; Nagelkerke $R^2 = 0.35$; correct classification = 71.2%) are also reported.
- Addressing confidence interval width: We acknowledge that the confidence interval (2.24–24.94) is relatively wide. This limitation is now addressed in the Discussion section, where we note that the wide CI likely reflects the relatively small sample size and the exploratory nature of this study. Importantly, the lower bound remains substantially above 1 (2.24), supporting the robustness of the finding.

Reviewer #2: Figure 1 needs a clearer explanation in the text

Answer: "Thank you for your comment regarding Figure 1. We have revised the manuscript to provide a clearer explanation of the figure in the text.

In the revised Results section, we now state:

'Based on the ICIQ-SF question "When does urine leak?", 16.44% of participants (n=12) reported no urine leakage. Among the 83.56% of participants who experienced some form of incontinence, the most frequently reported circumstances were: leakage when asleep (20.55%, n=15), leakage when coughing or sneezing (17.81%, n=13), leakage during physical activity (15.07%, n=11), leakage after finishing urination (9.59%, n=7), leakage before reaching the toilet (8.22%, n=6), constant leakage (6.85%, n=5), and leakage for no obvious reason (5.48%, n=4). The distribution of urinary incontinence patterns is illustrated in Figure 1.'

Additionally, we have expanded the figure caption to: *'Distribution of urinary incontinence patterns among nursing home residents (n=73). Based on ICIQ-SF item: "When does urine leak?" Values represent percentage of the total sample and number of participants.'*

We believe these revisions provide a clearer and more comprehensive explanation of Figure 1 as requested."

Reviewer #2: Tables are very dense and heavy on abbreviations

Answer: We have revised all tables to improve clarity and reduce density. Simplified table footnotes by removing redundant abbreviations and providing only essential information in a clear, concise format. Combined the unstandardized regression coefficient (B) and its 95% confidence interval into a single column (B [95% CI]) to streamline presentation and enhance readability.

Reviewer #2: UI and swallowing function can also be influenced by:BMI, Mobility, Comorbidities, Hydration status, Medication burden.If these variables are unavailable, please explicitly acknowledge residual confounding as a limitation. If some data exist (e.g., BMI), consider including them in an exploratory model.

Answer:

- Handgrip strength (sarcopenia): In response to reviewer comments regarding potential confounding by generalized muscle weakness and sarcopenia, we obtained handgrip strength data from institutional records—as these measurements are routinely collected every three months in our nursing home—and attempted to include this variable in our regression models as a proxy for sarcopenia. However, during diagnostic testing for multicollinearity, we encountered a significant statistical issue. The correlation matrix revealed an extremely high negative correlation between handgrip strength and gender (r = -.959), indicating that these two variables shared almost identical variance. Consequently, the Variance Inflation Factor (VIF) values for gender and handgrip strength were 12.55 and 12.54, respectively—far exceeding the recommended threshold of 5 [Craney and Surles 2002; O'brien 2007]. As noted by Craney and Surles (2002), VIF values above 5 indicate problematic multicollinearity that can distort regression coefficients, inflate standard errors, and compromise the reliability and validity of statistical models. Following established methodological recommendations [Kim 2009], researchers facing high multicollinearity have several options: remove one of the correlated variables, use ridge regression, or combine variables into a single index. Given that gender is a fundamental demographic variable with strong theoretical justification for inclusion, and given the exceptionally high correlation between handgrip strength and gender, we made the considered decision to exclude handgrip strength from the final models. This approach ensures stable, interpretable, and reliable regression results. We have now explicitly acknowledged in the Limitations section that, despite our attempt to include handgrip strength, sarcopenia could not be fully controlled for as a potential confounder due to these statistical constraints.

Reference: Craney, T. A., & Surles, J. G. (2002). Model-Dependent Variance Inflation Factor Cutoff Values. Quality Engineering, 14(3), 391–403. https://doi.org/10.1081/QEN-120001878

O'brien, R.M. A Caution Regarding Rules of Thumb for Variance Inflation Factors. Qual Quant 41, 673–690 (2007). https://doi.org/10.1007/s11135-006-9018-6

Kim, J. H. (2019). Multicollinearity and misleading statistical results. Korean journal of anesthesiology, 72(6), 558-569.

Correlations

		ICIQTotal	Presbyphagia	Age	Gender	handgrip
Pearson Correlation	ICIQTotal	1,000	,384	,087	,187	-,201
	Presbyphagia	,384	1,000	,255	-,036	,015
	Age	,087	,255	1,000	,033	-,036
	Gender	,187	-,036	,033	1,000	-,959
	handgrip	-,201	,015	-,036	-,959	1,000
Sig. (1-tailed)	ICIQTotal	.	,000	,232	,057	,044
	Presbyphagia	,000	.	,015	,380	,452
	Age	,232	,015	.	,390	,380
	Gender	,057	,380	,390	.	,000
	handgrip	,044	,452	,380	,000	.
N	ICIQTotal	73	73	73	73	73
	Presbyphagia	73	73	73	73	73
	Age	73	73	73	73	73
	Gender	73	73	73	73	73
	handgrip	73	73	73	73	73

Figure 1. Correlations (handgrip vs gender)

Coefficients[a]

Model		Unstandardized Coefficients		Standardized Coefficients	t	Sig.	95,0% Confidence Interval for B		Correlations			Collinearity Statistics	
		B	Std. Error	Beta			Lower Bound	Upper Bound	Zero-order	Partial	Part	Tolerance	VIF
1	(Constant)	4,274	12,041		,355	,724	-19,753	28,302					
	Presbyphagia	3,252	,937	,393	3,472	,001	1,383	5,121	,384	,388	,379	,928	1,077
	Age	-,011	,058	-,021	-,185	,854	-,126	,105	,087	-,022	-,020	,933	1,072
	Gender	,294	3,176	,036	,093	,927	-6,045	6,632	,187	,011	,010	,080	12,559
	handgrip	-,125	,279	-,173	-,448	,655	-,682	,432	-,201	-,054	-,049	,080	12,542

a. Dependent Variable: ICIQTotal

Figure 2. VIF value

- BMI: We chose not to include BMI in the final models for several methodological reasons. First, to avoid increasing Type II error risk: Adding BMI as an additional covariate would increase the number of statistical tests, thereby lowering the significance threshold when applying multiple comparison corrections (e.g., Bonferroni). This could obscure truly meaningful associations. Second, with a sample size of 73, including too many covariates risks overfitting and unstable estimates (Pergener, 1998). Following principles of parsimonious modeling, we prioritized variables with stronger theoretical justification: presbyphagia, age, and gender. Third, preliminary bivariate analyses showed no significant association between BMI and our primary outcomes, further supporting this decision. We believe this balanced approach maintains statistical power while appropriately addressing confounding.

Referance: “Perneger TV. What's wrong with Bonferroni adjustments. BMJ. 1998 Apr 18;316(7139):1236-8. doi: 10.1136/bmj.316.7139.1236. PMID: 9553006; PMCID: PMC1112991.

- Mobility, comorbidities, and medication burden: These potential confounders were addressed through rigorous a priori exclusion criteria, which we believe strengthens our methodological approach.

  Mobility: Individuals with a history of falls or requiring ambulation assistance were excluded (criterion #2), ensuring a functionally independent sample with preserved mobility.

  Comorbidities: We excluded individuals with neurological/psychiatric diseases (criterion #3), conditions affecting swallowing/UI mechanisms (criterion #4), and acute systemic illnesses (criterion #6), thereby reducing confounding from major comorbidities.

  Medication burden: Regular use of medications affecting urinary function or cognition (e.g., diuretics, anticholinergics) was an exclusion criterion (criterion #5), eliminating pharmacological confounding from these classes.
- Hydration : We acknowledge that hydration status was not directly measured in this study, and we have now added this as an explicit limitation in the revised manuscript. Future studies should incorporate objective measures of hydration status to further clarify its potential role in the presbyphagia-UI relationship.

Response to Reviewer #3

Reviewer #3: This is an original study that raises an interesting but conceptually challenging question. The primary aim is to demonstrate an association between presbyphagia and urinary incontinence. While such an association is statistically demonstrated, causation is not established, and the clinical or practical implications of this finding remain unclear. The results are statistically significant but conceptually weak in terms of mechanistic or clinical interpretation.

Answer: We thank the reviewer for these important observations regarding causal inference and clinical interpretation. We fully agree that this cross-sectional study cannot establish causality, and we have carefully revised the manuscript throughout to reflect this.

Specifically, we have: Replaced all causal language (e.g., "predicts," "leads to") with associative terms ("is associated with," "is linked to") throughout the manuscript. Softened all clinical implications, presenting findings as hypothesis-generating rather than practice-changing. For example, statements suggesting routine screening have been revised to indicate that presbyphagia "may warrant clinical attention" in comprehensive geriatric assessment. Added discussion of potential mechanisms (e.g., autonomic nervous system dysfunction, shared neuromuscular pathways) while explicitly noting that these remain speculative and require confirmation in future studies.

Reviewer #3: "Several methodological concerns merit attention. Urinary incontinence is not a single clinical entity; stress and urgency incontinence have distinct mechanisms and etiologies. In the present study, incontinence appears to be treated as a unified condition and linked to presbyphagia as a single construct, which may oversimplify the clinical reality."

Answer: We thank the reviewer for this important observation. While our primary regression analyses treated UI as a unified condition (using ICIQ-SF total scores), we did collect data on UI subtypes through the ICIQ-SF item "When does urine leak?" As presented in Figure 1, the distribution was: stress-pattern UI (cough/sneeze + physical activity) in 24 participants (32.9%), urgency-pattern UI in 6 participants (8.2%), other patterns in 31 participants (42.5%), and no leakage in 12 participants (16.4%). Due to the small number of participants with urgency UI (n=6), subtype-specific statistical analyses were not feasible. We have now acknowledged this as a limitation (Limitations section, last point) and recommend that future studies with larger samples investigate subtype-specific associations.

Reviewer #3: In addition, the sample size is small and drawn from a single population, limiting generalisability.

Answer: We fully concur with the reviewer that the sample size (n=73) is modest and drawn from a single population, which limits generalizability. However, it is important to note that this final sample was derived from an initial pool of 298 residents after applying rigorous exclusion criteria to ensure a homogeneous, functionally independent population without major confounders. A post-hoc power analysis using the observed effect size ($f^2 = 0.17$) from our study data confirmed that the study achieved 97% statistical power at a 0.05 significance level. This indicates that despite the modest sample size and the effect size being lower than initially anticipated, our sample was adequately powered to detect the observed associations, supporting the internal validity of the findings.

We have revised the manuscript throughout to emphasize the exploratory, hypothesis-generating nature of this study. The Limitations section now explicitly acknowledges the limited generalizability due to the single-center design and modest sample size, while the Conclusion emphasizes that our study provides a foundation for future research with larger, more diverse cohorts rather than definitive clinical recommendations.

Reviewer #3: Overall, the study is best interpreted as exploratory and association-based, rather than as a hypothesis-confirming or clinically directive investigation.

Answer: We fully concur with the reviewer. We have revised the manuscript throughout to emphasize the exploratory, hypothesis-generating nature of this study. All causal language has been replaced with associative terminology, and clinical implications have been softened to reflect the preliminary nature of the findings. The Conclusion now explicitly states that our study provides a foundation for future research rather than definitive clinical recommendations.. We have carefully reviewed the entire manuscript and replaced all instances of causal language (e.g., "predicts," "affects," "leads to") with appropriate associative terms such as "is associated with," "is related to," and "is linked to" throughout the Abstract, Results, Discussion, and Conclusion sections.

Reviewer #3: "The main strengths of the manuscript include the use of validated questionnaires, clear reporting of regression coefficients and correlations, and the absence of obvious internal data inconsistencies. However, it would be more appropriate to replace the term "predicts" with "is associated with" throughout the manuscript when describing regression results."

Answer: Thank you for your positive feedback regarding the use of validated questionnaires and clear reporting of statistical results. We appreciate your constructive suggestion regarding terminology. In response, we have carefully reviewed the entire manuscript and replaced all instances of causal language (e.g., "predicts," "affects," "leads to") with appropriate associative terms such as "is associated with," "is related to," and "is linked to" throughout the text. We believe this revision more accurately reflects the cross-sectional nature of our study and aligns with your recommendation. Thank you for helping us improve the precision of our language.

Reviwer 3: "Although I am not a statistician, the manuscript would benefit from formal statistical review. In particular, there appears to be a multiple comparisons issue, with approximately 30 hypothesis tests conducted without correction."

Answer: "We have carefully considered this and implemented a balanced, theoretically justified approach:

- For the 10 KHQ subscales: These represent a family of related outcomes measuring the same underlying construct (incontinence-specific quality of life). We applied Bonferroni correction within this domain only ($\alpha = 0.05/10 = 0.005$).
- For the ICIQ-SF (UI) analysis: This tests a separate, independent hypothesis (presbyphagia-UI association) distinct from the QOL outcomes. Applying Bonferroni across unrelated research questions would be excessively conservative and increase Type II error risk, potentially obscuring real associations (Perneger, 1998; Rothman, 1990). Therefore, we did not apply correction to this primary hypothesis test.
- We have added a short sentence to the Statistical Analysis section to address this correction.

We believe this nuanced approach appropriately balances the risks of both Type I and Type II eros while maintaining scientific rigor."

Referance: "Perneger TV. What's wrong with Bonferroni adjustments. BMJ. 1998 Apr 18;316(7139):1236-8. doi: 10.1136/bmj.316.7139.1236. PMID: 9553006; PMCID: PMC1112991."

Reviwer 3: "Additionally, a logistic regression analysis is implied by the reporting of an odds ratio (OR = 4.61), but the corresponding model and confidence intervals are not presented."

Answer: In the revised manuscript, we have now clearly described the binary logistic regression analysis in the Statistical Analysis section and presented the adjusted odds ratios with their 95% confidence intervals. For the association between presbyphagia and UI, the adjusted odds ratio is AOR = 7.47 (95% CI: 2.24–24.94, p = 0.001) , adjusted for age and gender. Additionally, male gender was independently associated with higher odds of UI presence (AOR = 5.16, 95% CI: 1.53–17.42, p = 0.008) . The initial OR value of 4.61 was a preliminary estimate and has been replaced with these more robust analyses. We have also added a new table presenting the full binary logistic regression results, including all covariates and their corresponding odds ratios, confidence intervals, and p-values.

Reviwer 3: Conceptual overlap: Presbyphagia is defined using EAT-10, a symptom-based instrument, while outcomes include multiple symptom-based and quality-of-life scales (ICIQ-SF, KHQ). The observed associations may therefore reflect overall symptom burden, frailty, or response bias rather than a specific link between swallowing function and urinary continence. This limitation should be explicitly acknowledged, and conclusions reframed accordingly.

Answer: We acknowledge that both presbyphagia and UI outcomes were assessed using symptom-based questionnaires. Therefore, the observed associations may partially reflect overall symptom burden or response bias rather than a specific physiological link.

In response to this valuable feedback, we have:

- Expanded our existing limitations statement (formerly "Third, self-reported data are susceptible to recall and response biases") to now explicitly read: "Third, self-reported data are susceptible to recall and response biases, as both presbyphagia and UI outcomes were assessed using symptom-based questionnaires."
- Added a corresponding statement in the Conclusion section to ensure interpretation reflects this limitation: "Additionally, both presbyphagia and UI outcomes were assessed using symptom-based questionnaires, which introduces the possibility that the observed associations may partially reflect overall symptom burden or response bias rather than a specific physiological link."

These revisions ensure that this limitation is both explicitly acknowledged and consistently reflected in our interpretation of the findings. We believe this addresses the reviewer's concern appropriately.

Reviwer 3: Causality: At several points, the manuscript implies a directional or causal relationship. These statements should be revised to consistently emphasise association only.

Answer: In response, we have carefully reviewed the entire manuscript and replaced all causal language (e.g., "predicts," "affects," "leads to") with appropriate associative terms such as "is associated with," "is related to," and "is linked to" throughout the Abstract, Results, Discussion, and Conclusion sections. The manuscript now consistently emphasizes association rather than causation.

Reviwer 3: Clinical relevance: The variance explained by the regression models is modest (R^2 approximately 0.10–0.20). Statements suggesting clinical screening, diagnostic implications, or practice change should therefore be avoided.

Answer: We have softened all clinical implications throughout the manuscript. Statements suggesting clinical screening or practice change have been revised to reflect the exploratory nature of our findings. The Conclusion now emphasizes that our study provides a foundation for future research rather than definitive clinical recommendations.

Reviwer 3: If available, comorbidity or frailty measures should be considered as potential confounders.

Answer: Regarding comorbidities, our study design addressed this through rigorous a priori exclusion criteria. Participants with known neurological or psychiatric diseases (e.g., stroke, Parkinson's disease, dementia), conditions directly affecting swallowing or UI mechanisms, and acute or uncontrolled systemic illnesses were excluded from the study. This approach resulted in the exclusion of 225 out of 298 initially assessed individuals, yielding a final sample of 73 functionally independent older adults with minimal major comorbidities. We obtained handgrip strength measurements—which are routinely collected in our institution every three months—from the institutional database and attempted to incorporate them into our regression model as a proxy measure for sarcopenia. However, during diagnostic testing for multicollinearity, we encountered a critical statistical issue: an extremely high correlation between handgrip strength and gender ($r = -.959$), resulting in Variance Inflation Factor (VIF) values of 12.55 and 12.54, respectively. These values substantially exceeded the recommended threshold of 5 (Craney and Surles 2002; O'Brien 2007), indicating that including both variables would have compromised the reliability and validity of our model.

Following established methodological recommendations (Kim 2019), we chose to exclude handgrip strength from our analyses, as its high correlation with gender made simultaneous inclusion statistically problematic. This decision ensured the consistency and reliability of our regression models. As you recommended, we have now explicitly stated in the Limitations section that, despite our attempt to include handgrip strength, sarcopenia could not be adequately controlled for as a potential confounder.

Correlations

		ICIQTotal	Presbyphagia	Age	Gender	handgrip
Pearson Correlation	ICIQTotal	1,000	,384	,087	,187	-,201
	Presbyphagia	,384	1,000	,255	-,036	,015
	Age	,087	,255	1,000	,033	-,036
	Gender	,187	-,036	,033	1,000	-,959
	handgrip	-,201	,015	-,036	-,959	1,000
Sig. (1-tailed)	ICIQTotal	.	,000	,232	,057	,044
	Presbyphagia	,000	.	,015	,380	,452
	Age	,232	,015	.	,390	,380
	Gender	,057	,380	,390	.	,000
	handgrip	,044	,452	,380	,000	.
N	ICIQTotal	73	73	73	73	73
	Presbyphagia	73	73	73	73	73
	Age	73	73	73	73	73
	Gender	73	73	73	73	73
	handgrip	73	73	73	73	73

Figure 1. Correlations (handgrip vs gender)

Coefficients[a]

Model		Unstandardized Coefficients		Standardized Coefficients	t	Sig.	95,0% Confidence Interval for B		Correlations			Collinearity Statistics	
		B	Std. Error	Beta			Lower Bound	Upper Bound	Zero-order	Partial	Part	Tolerance	VIF
1	(Constant)	4,274	12,041		,355	,724	-19,753	28,302					
	Presbyphagia	3,252	,937	,393	3,472	,001	1,383	5,121	,384	,388	,379	,928	1,077
	Age	-,011	,058	-,021	-,185	,854	-,126	,105	,087	-,022	-,020	,933	1,072
	Gender	,294	3,176	,036	,093	,927	-6,045	6,632	,187	,011	,010	,080	12,559
	handgrip	-,125	,279	-,173	-,448	,655	-,682	,432	-,201	-,054	-,049	,080	12,542

a. Dependent Variable: ICIQTotal

Figure 2. VIF value

Reference:

Craney, T. A., & Surles, J. G. (2002). Model-Dependent Variance Inflation Factor Cutoff Values. *Quality Engineering*, *14*(3), 391–403. https://doi.org/10.1081/QEN-120001878

O'brien, R.M. A Caution Regarding Rules of Thumb for Variance Inflation Factors. *Qual Quant* **41**, 673–690 (2007). https://doi.org/10.1007/s11135-006-9018-6

Kim, J. H. (2019). Multicollinearity and misleading statistical results. *Korean journal of anesthesiology*, *72*(6), 558-569.

Reviwer 3: Recruitment setting and exclusion criteria should be clarified.

Answer: The recruitment setting and exclusion criteria are already detailed in the Methods section under "2.1 Study design and setting" and "2.2 Participants and Sample Size". As described, participants were recruited from two nursing homes in Isparta, Turkey, between December 23-30, 2024. A total of 298 residents were assessed for eligibility, and 225 were excluded based on the predefined exclusion criteria listed in the manuscript. Although we recorded the exact numbers excluded for each criterion, we chose not to include these detailed frequencies in the manuscript to avoid lengthening the text unnecessarily. The remaining 73 participants constituted the final sample. We believe this provides a comprehensive and transparent description of the participant selection process.

If the editor or reviewers feel that including these detailed exclusion numbers would strengthen the manuscript, we are happy to add them.

Reviwer 3: In conclusion, the study demonstrates a statistical association between presbyphagia and urinary incontinence. Older adults classified as having presbyphagia score worse on ICIQ-SF, KHQ, and quality-of-life questionnaires. These findings are compatible with broader constructs such as frailty, comorbidity, and symptom burden. However, the study does not establish a causal relationship, does not identify a shared physiological mechanism, and does not justify clinical screening for urinary incontinence based on presbyphagia alone. The manuscript may be considered for publication provided the above issues are adequately addressed and the exploratory nature of the findings is clearly acknowledged.

Answer: We fully agree that this study demonstrates a statistical association rather than a causal relationship. In response, we have emphasized the exploratory nature of our findings throughout the manuscript, replaced all causal language with associative terminology, softened clinical implications, and expanded the Limitations section to address the issues raised. We believe these revisions adequately address the reviewer's concerns.
