# Supplementary material for: The association of presbyphagia with urinary incontinence and Incontinence-related quality of life in nursing home residents: A cross-sectional study
Source: PLoS One. 2026 Jun 4;21(6):e0350933. doi: 10.1371/journal.pone.0350933 (PMC13235875; doi:10.1371/journal.pone.0350933)
Supplement: Attachment — Submitted filename: Response to Reviewers.docx [file pone.0350933.s001.docx]

**Response to Reviewer #1**

**Reviewer #1:** “…However, there is a central point that deserves greater attention: presbyphagia and urinary incontinence frequently coexist because both emerge from the same pathophysiological context. This shared set of determinants creates a scenario in which any observed association between the two conditions is likely explained by confounding factors, especially sarcopenia. Since this important confounder was not analysed, even statistical adjustments cannot fully address this limitation, which compromises the interpretation of the results. Therefore, I recommend restructuring the text so that the findings related to the dependent variables—presbyphagia and urinary incontinence—are presented in relation to quality‑of‑life measures independently, avoiding causal inferences between presbyphagia and incontinence.”

**Answer:** Thank you for your insightful comment regarding the shared pathophysiological background of presbyphagia and urinary incontinence, and the potential confounding role of sarcopenia. You have raised a very important point that we had not initially considered. In response to your valuable feedback, we recognized the need to control for sarcopenia in our analysis. Since handgrip strength measurements are routinely collected in our institution every three months and recorded in the institutional database, we obtained the values corresponding to the study period and attempted to incorporate them into our regression model as a proxy measure for sarcopenia. However, during the diagnostic process for multicollinearity, we encountered a critical statistical issue: a very high correlation between handgrip strength and gender (r = -.959) (Figure 1), resulting in Variance Inflation Factor (VIF) values of 12.55 and 12.54 (Figure 2), respectively. Since VIF values significantly exceeded the recommended threshold of 5 (Craney and Surles 2002), including both variables would have compromised the reliability and validity of our model. Consequently, despite our attempt to address your concern by obtaining and testing handgrip strength data, we had to exclude it from the final analysis due to these statistical limitations.

As noted by O'Brien (2007), when VIF values exceed recommended thresholds, researchers commonly address multicollinearity by removing one of the correlated variables, using ridge regression, or combining variables into a single index. Similarly, Kim (2019) emphasizes that excluding multicollinear predictors is an appropriate approach to obtain stable and reliable regression models. Following these established methodological recommendations, we chose to exclude handgrip strength from our analyses, as its high correlation with gender (r = -.959; VIF > 5) made simultaneous inclusion of both variables statistically problematic. This decision ensured the consistency and reliability of our regression models. As you recommended, we have now explicitly stated in the **Limitations section** that, although we attempted to include handgrip strength, sarcopenia could not be adequately controlled for as a potential confounder. Due to these changes and to avoid causal inferences, we have restructured the text.

Figure 1. Correlations (handgrip vs gender)

Figure 2. VIF value

Reference:

Craney, T. A., & Surles, J. G. (2002). Model-Dependent Variance Inflation Factor Cutoff Values. *Quality Engineering*, *14*(3), 391–403. https://doi.org/10.1081/QEN-120001878

O’brien, R.M. A Caution Regarding Rules of Thumb for Variance Inflation Factors. *Qual Quant* **41**, 673–690 (2007). https://doi.org/10.1007/s11135-006-9018-6

Kim, J. H. (2019). Multicollinearity and misleading statistical results. *Korean journal of anesthesiology*, *72*(6), 558-569.

**Response to Reviewer #2**

**Reviewer #2:** “The abstract is slightly long and statistically dense. Abstract could be made little simpler..”

**Answer:** Thank you for your valuable feedback. We have revised the abstract to be more concise and less statistically dense. We believe this version is simpler and more accessible to readers.

**Reviewer #2:** “This is a cross-sectional study, but at several places the wording sounds like presbyphagia leads to urinary incontinence. Please be careful with language-Say “associated with”, not “predicts”, “affects”, or “should be screened as standard practice”. The conclusions and clinical suggestions should be more cautious and framed as hypothesis-generating, not practice-changing.”

**Answer:** a) Thank you for your insightful comment regarding the language used in our manuscript. You are absolutely right that in a cross-sectional study, terminology should reflect associations rather than causal relationships. Some of these wording issues arose during the translation process from our native language to English. We have now carefully revised the manuscript accordingly. For example:

Original: "Multiple linear analysis was performed to predict the UI dependent variables using the independent variable of presence/absence of presbyphagia, age and gender."

Revised: "Multiple linear regression analysis was conducted to examine the association between UI and the independent variables: presence/absence of presbyphagia, age, and gender."

b) In response to your comment, we have carefully revised the conclusion and discussion section accordingly. The overall tone of the conclusion now reflects a more cautious and hypothesis-generating perspective, highlighting the need for future longitudinal and interventional studies to confirm our findings.

**Reviewer #2:** Definition of presbyphagia needs clearer justification

**Answer:** We have substantially revised the introduction to provide a clearer, more comprehensive definition and justification of presbyphagia. The revised text now:

- Provides a precise, evidence-based definition of presbyphagia (structural, physiological, and innervational alterations)
- Specifies the underlying mechanisms (brain atrophy, neurological deterioration, muscle loss)
- Maintains connection to clinical symptoms with appropriate citations

We believe this revision addresses the reviewer's concern by clearly justifying what presbyphagia is and how it develops.

**Reviewer #2:** statistical analysis: The logistic regression model used to calculate the odds ratio is not clearly described Confidence intervals for the odds ratio are missing. Clearly state which analysis was used for the odds ratio. Report adjusted OR with 95% CI. Be consistent in how UI is analysed and interpreted

**Answer:** We thank the reviewers for their valuable comments regarding the odds ratio calculation and presentation. We have carefully addressed these points as follows:

- Removal of unclear odds ratio: The previous version of the manuscript included a statement: *'Participants were classified as having/not having UI according to the ICIQ-SF cut-off value and an odds ratio of 4.61 was calculated.'* We agree with the reviewers that this statement was insufficiently detailed, lacking information about the statistical method used, confidence intervals, and adjustment for confounders. This unclear statement has been removed entirely from the revised manuscript.
- Proper logistic regression analysis: In its place, we have now conducted and reported a binary logistic regression analysis following standard methodological guidelines. In the Statistical Analysis section, we clearly describe:

Variable coding: presbyphagia (0 = non-presbyphagia, 1 = presbyphagia), UI presence (ICIQ-SF ≥6; 0 = no UI, 1 = UI present)

Covariates: age and gender

Exclusion of handgrip strength due to high multicollinearity with gender (VIF > 5, r = -0.96)

- Complete reporting of results: In the Results section, we now present the adjusted odds ratio with its 95% confidence interval: OR = 7.47 (95% CI: 2.24–24.94, p = 0.001). A detailed table (Table 3) following simplified APA format now includes B coefficients, standard errors, Wald statistics, p-values, odds ratios, and confidence intervals for all variables. Model fit statistics (χ² = 21.53, df = 3, p < 0.001; Cox & Snell R² = 0.26; Nagelkerke R² = 0.35; correct classification = 71.2%) are also reported.
- Addressing confidence interval width: We acknowledge that the confidence interval (2.24–24.94) is relatively wide. This limitation is now addressed in the Discussion section, where we note that the wide CI likely reflects the relatively small sample size and the exploratory nature of this study. Importantly, the lower bound remains substantially above 1 (2.24), supporting the robustness of the finding.

**Reviewer #2:** Figure 1 needs a clearer explanation in the text

**Answer:** "Thank you for your comment regarding Figure 1. We have revised the manuscript to provide a clearer explanation of the figure in the text.

In the revised Results section, we now state:

*'Based on the ICIQ-SF question "When does urine leak?", 16.44% of participants (n=12) reported no urine leakage. Among the 83.56% of participants who experienced some form of incontinence, the most frequently reported circumstances were: leakage when asleep (20.55%, n=15), leakage when coughing or sneezing (17.81%, n=13), leakage during physical activity (15.07%, n=11), leakage after finishing urination (9.59%, n=7), leakage before reaching the toilet (8.22%, n=6), constant leakage (6.85%, n=5), and leakage for no obvious reason (5.48%, n=4). The distribution of urinary incontinence patterns is illustrated in Figure 1.'*

Additionally, we have expanded the figure caption to: *'Distribution of urinary incontinence patterns among nursing home residents (n=73). Based on ICIQ-SF item: "When does urine leak?" Values represent percentage of the total sample and number of participants.'*

We believe these revisions provide a clearer and more comprehensive explanation of Figure 1 as requested."

**Reviewer #2:** Tables are very dense and heavy on abbreviations

**Answer:** We have revised all tables to improve clarity and reduce density. Simplified table footnotes by removing redundant abbreviations and providing only essential information in a clear, concise format. Combined the unstandardized regression coefficient (B) and its 95% confidence interval into a single column (B [95% CI]) to streamline presentation and enhance readability.

**Reviewer #2:** UI and swallowing function can also be influenced by:BMI, Mobility, Comorbidities, Hydration status, Medication burden.If these variables are unavailable, please explicitly acknowledge residual confounding as a limitation. If some data exist (e.g., BMI), consider including them in an exploratory model.

**Answer:**

- Handgrip strength (sarcopenia): In response to reviewer comments regarding potential confounding by generalized muscle weakness and sarcopenia, we obtained handgrip strength data from institutional records—as these measurements are routinely collected every three months in our nursing home—and attempted to include this variable in our regression models as a proxy for sarcopenia. However, during diagnostic testing for multicollinearity, we encountered a significant statistical issue. The correlation matrix revealed an extremely high negative correlation between handgrip strength and gender (r = -.959), indicating that these two variables shared almost identical variance. Consequently, the Variance Inflation Factor (VIF) values for gender and handgrip strength were 12.55 and 12.54, respectively—far exceeding the recommended threshold of 5 [Craney and Surles 2002; O’brien 2007]. As noted by Craney and Surles (2002), VIF values above 5 indicate problematic multicollinearity that can distort regression coefficients, inflate standard errors, and compromise the reliability and validity of statistical models. Following established methodological recommendations [Kim 2009], researchers facing high multicollinearity have several options: remove one of the correlated variables, use ridge regression, or combine variables into a single index. Given that gender is a fundamental demographic variable with strong theoretical justification for inclusion, and given the exceptionally high correlation between handgrip strength and gender, we made the considered decision to exclude handgrip strength from the final models. This approach ensures stable, interpretable, and reliable regression results. We have now explicitly acknowledged in the Limitations section that, despite our attempt to include handgrip strength, sarcopenia could not be fully controlled for as a potential confounder due to these statistical constraints.

Reference: Craney, T. A., & Surles, J. G. (2002). Model-Dependent Variance Inflation Factor Cutoff Values. Quality Engineering, 14(3), 391–403. https://doi.org/10.1081/QEN-120001878

O’brien, R.M. A Caution Regarding Rules of Thumb for Variance Inflation Factors. Qual Quant 41, 673–690 (2007). https://doi.org/10.1007/s11135-006-9018-6

Kim, J. H. (2019). Multicollinearity and misleading statistical results. Korean journal of anesthesiology, 72(6), 558-569.

Figure 1. Correlations (handgrip vs gender)

Figure 2. VIF value

- BMI: We chose not to include BMI in the final models for several methodological reasons. First, to avoid increasing Type II error risk: Adding BMI as an additional covariate would increase the number of statistical tests, thereby lowering the significance threshold when applying multiple comparison corrections (e.g., Bonferroni). This could obscure truly meaningful associations. Second, with a sample size of 73, including too many covariates risks overfitting and unstable estimates (Pergener, 1998). Following principles of parsimonious modeling, we prioritized variables with stronger theoretical justification: presbyphagia, age, and gender. Third, preliminary bivariate analyses showed no significant association between BMI and our primary outcomes, further supporting this decision. We believe this balanced approach maintains statistical power while appropriately addressing confounding.

Referance: “Perneger TV. What's wrong with Bonferroni adjustments. BMJ. 1998 Apr 18;316(7139):1236-8. doi: 10.1136/bmj.316.7139.1236. PMID: 9553006; PMCID: PMC1112991.

- Mobility, comorbidities, and medication burden: These potential confounders were addressed through rigorous a priori exclusion criteria, which we believe strengthens our methodological approach.

Mobility: Individuals with a history of falls or requiring ambulation assistance were excluded (criterion #2), ensuring a functionally independent sample with preserved mobility.

Comorbidities: We excluded individuals with neurological/psychiatric diseases (criterion #3), conditions affecting swallowing/UI mechanisms (criterion #4), and acute systemic illnesses (criterion #6), thereby reducing confounding from major comorbidities.

Medication burden: Regular use of medications affecting urinary function or cognition (e.g., diuretics, anticholinergics) was an exclusion criterion (criterion #5), eliminating pharmacological confounding from these classes.

- Hydration : We acknowledge that hydration status was not directly measured in this study, and we have now added this as an explicit limitation in the revised manuscript. Future studies should incorporate objective measures of hydration status to further clarify its potential role in the presbyphagia-UI relationship.

**Response to Reviewer #3**

**Reviewer #3:** This is an original study that raises an interesting but conceptually challenging question. The primary aim is to demonstrate an association between presbyphagia and urinary incontinence. While such an association is statistically demonstrated, causation is not established, and the clinical or practical implications of this finding remain unclear. The results are statistically significant but conceptually weak in terms of mechanistic or clinical interpretation.

**Answer:** We thank the reviewer for these important observations regarding causal inference and clinical interpretation. We fully agree that this cross-sectional study cannot establish causality, and we have carefully revised the manuscript throughout to reflect this.

Specifically, we have: Replaced all causal language (e.g., "predicts," "leads to") with associative terms ("is associated with," "is linked to") throughout the manuscript. Softened all clinical implications, presenting findings as hypothesis-generating rather than practice-changing. For example, statements suggesting routine screening have been revised to indicate that presbyphagia "may warrant clinical attention" in comprehensive geriatric assessment. Added discussion of potential mechanisms (e.g., autonomic nervous system dysfunction, shared neuromuscular pathways) while explicitly noting that these remain speculative and require confirmation in future studies.

**Reviewer #3: “**Several methodological concerns merit attention. Urinary incontinence is not a single clinical entity; stress and urgency incontinence have distinct mechanisms and etiologies. In the present study, incontinence appears to be treated as a unified condition and linked to presbyphagia as a single construct, which may oversimplify the clinical reality.”

**Answer:** We thank the reviewer for this important observation. While our primary regression analyses treated UI as a unified condition (using ICIQ-SF total scores), we did collect data on UI subtypes through the ICIQ-SF item "When does urine leak?" As presented in Figure 1, the distribution was: stress-pattern UI (cough/sneeze + physical activity) in 24 participants (32.9%), urgency-pattern UI in 6 participants (8.2%), other patterns in 31 participants (42.5%), and no leakage in 12 participants (16.4%). Due to the small number of participants with urgency UI (n=6), subtype-specific statistical analyses were not feasible. We have now acknowledged this as a limitation (Limitations section, last point) and recommend that future studies with larger samples investigate subtype-specific associations.

**Reviewer #3:** In addition, the sample size is small and drawn from a single population, limiting generalisability.

**Answer:** We fully concur with the reviewer that the sample size (n=73) is modest and drawn from a single population, which limits generalizability. However, it is important to note that this final sample was derived from an initial pool of 298 residents after applying rigorous exclusion criteria to ensure a homogeneous, functionally independent population without major confounders. A post-hoc power analysis using the observed effect size (f² = 0.17) from our study data confirmed that the study achieved 97% statistical power at a 0.05 significance level. This indicates that despite the modest sample size and the effect size being lower than initially anticipated, our sample was adequately powered to detect the observed associations, supporting the internal validity of the findings.

We have revised the manuscript throughout to emphasize the exploratory, hypothesis-generating nature of this study. The Limitations section now explicitly acknowledges the limited generalizability due to the single-center design and modest sample size, while the Conclusion emphasizes that our study provides a foundation for future research with larger, more diverse cohorts rather than definitive clinical recommendations.

**Reviewer #3:** Overall, the study is best interpreted as exploratory and association-based, rather than as a hypothesis-confirming or clinically directive investigation.

**Answer:** We fully concur with the reviewer. We have revised the manuscript throughout to emphasize the exploratory, hypothesis-generating nature of this study. All causal language has been replaced with associative terminology, and clinical implications have been softened to reflect the preliminary nature of the findings. The Conclusion now explicitly states that our study provides a foundation for future research rather than definitive clinical recommendations.. We have carefully reviewed the entire manuscript and replaced all instances of causal language (e.g., "predicts," "affects," "leads to") with appropriate associative terms such as "is associated with," "is related to," and "is linked to" throughout the Abstract, Results, Discussion, and Conclusion sections.

**Reviewer #3: “**The main strengths of the manuscript include the use of validated questionnaires, clear reporting of regression coefficients and correlations, and the absence of obvious internal data inconsistencies. However, it would be more appropriate to replace the term “predicts” with “is associated with” throughout the manuscript when describing regression results.”

**Answer:** Thank you for your positive feedback regarding the use of validated questionnaires and clear reporting of statistical results. We appreciate your constructive suggestion regarding terminology. In response, we have carefully reviewed the entire manuscript and replaced all instances of causal language (e.g., "predicts," "affects," "leads to") with appropriate associative terms such as "is associated with," "is related to," and "is linked to" throughout the text. We believe this revision more accurately reflects the cross-sectional nature of our study and aligns with your recommendation. Thank you for helping us improve the precision of our language.

**Reviwer 3:** “Although I am not a statistician, the manuscript would benefit from formal statistical review. In particular, there appears to be a multiple comparisons issue, with approximately 30 hypothesis tests conducted without correction.”

**Answer:** “We have carefully considered this and implemented a balanced, theoretically justified approach:

- For the 10 KHQ subscales: These represent a family of related outcomes measuring the same underlying construct (incontinence-specific quality of life). We applied Bonferroni correction within this domain only (α = 0.05/10 = 0.005).
- For the ICIQ-SF (UI) analysis: This tests a separate, independent hypothesis (presbyphagia-UI association) distinct from the QOL outcomes. Applying Bonferroni across unrelated research questions would be excessively conservative and increase Type II error risk, potentially obscuring real associations (Perneger, 1998; Rothman, 1990). Therefore, we did not apply correction to this primary hypothesis test.
- We have added a short sentence to the Statistical Analysis section to address this correction.

We believe this nuanced approach appropriately balances the risks of both Type I and Type II eros while maintaining scientific rigor.”

Referance: “Perneger TV. What's wrong with Bonferroni adjustments. BMJ. 1998 Apr 18;316(7139):1236-8. doi: 10.1136/bmj.316.7139.1236. PMID: 9553006; PMCID: PMC1112991.”

**Reviwer 3: “**Additionally, a logistic regression analysis is implied by the reporting of an odds ratio (OR = 4.61), but the corresponding model and confidence intervals are not presented.”

**Answer:** In the revised manuscript, we have now clearly described the binary logistic regression analysis in the Statistical Analysis section and presented the adjusted odds ratios with their 95% confidence intervals. For the association between presbyphagia and UI, the adjusted odds ratio is AOR = 7.47 (95% CI: 2.24–24.94, p = 0.001) , adjusted for age and gender. Additionally, male gender was independently associated with higher odds of UI presence (AOR = 5.16, 95% CI: 1.53–17.42, p = 0.008) . The initial OR value of 4.61 was a preliminary estimate and has been replaced with these more robust analyses. We have also added a new table presenting the full binary logistic regression results, including all covariates and their corresponding odds ratios, confidence intervals, and p-values.

**Reviwer 3:** Conceptual overlap: Presbyphagia is defined using EAT-10, a symptom-based instrument, while outcomes include multiple symptom-based and quality-of-life scales (ICIQ-SF, KHQ). The observed associations may therefore reflect overall symptom burden, frailty, or response bias rather than a specific link between swallowing function and urinary continence. This limitation should be explicitly acknowledged, and conclusions reframed accordingly.

**Answer:** We acknowledge that both presbyphagia and UI outcomes were assessed using symptom-based questionnaires. Therefore, the observed associations may partially reflect overall symptom burden or response bias rather than a specific physiological link.

In response to this valuable feedback, we have:

- Expanded our existing limitations statement (formerly "Third, self-reported data are susceptible to recall and response biases") to now explicitly read: "Third, self-reported data are susceptible to recall and response biases, as both presbyphagia and UI outcomes were assessed using symptom-based questionnaires."
- Added a corresponding statement in the Conclusion section to ensure interpretation reflects this limitation: "Additionally, both presbyphagia and UI outcomes were assessed using symptom-based questionnaires, which introduces the possibility that the observed associations may partially reflect overall symptom burden or response bias rather than a specific physiological link."

These revisions ensure that this limitation is both explicitly acknowledged and consistently reflected in our interpretation of the findings. We believe this addresses the reviewer's concern appropriately.

**Reviwer 3:** Causality: At several points, the manuscript implies a directional or causal relationship. These statements should be revised to consistently emphasise association only.

**Answer:** In response, we have carefully reviewed the entire manuscript and replaced all causal language (e.g., "predicts," "affects," "leads to") with appropriate associative terms such as "is associated with," "is related to," and "is linked to" throughout the Abstract, Results, Discussion, and Conclusion sections. The manuscript now consistently emphasizes association rather than causation.

**Reviwer 3:** Clinical relevance: The variance explained by the regression models is modest (R² approximately 0.10–0.20). Statements suggesting clinical screening, diagnostic implications, or practice change should therefore be avoided.

**Answer:** We have softened all clinical implications throughout the manuscript. Statements suggesting clinical screening or practice change have been revised to reflect the exploratory nature of our findings. The Conclusion now emphasizes that our study provides a foundation for future research rather than definitive clinical recommendations.

**Reviwer 3:** If available, comorbidity or frailty measures should be considered as potential confounders.

**Answer:** Regarding comorbidities, our study design addressed this through rigorous a priori exclusion criteria. Participants with known neurological or psychiatric diseases (e.g., stroke, Parkinson's disease, dementia), conditions directly affecting swallowing or UI mechanisms, and acute or uncontrolled systemic illnesses were excluded from the study. This approach resulted in the exclusion of 225 out of 298 initially assessed individuals, yielding a final sample of 73 functionally independent older adults with minimal major comorbidities. We obtained handgrip strength measurements—which are routinely collected in our institution every three months—from the institutional database and attempted to incorporate them into our regression model as a proxy measure for sarcopenia. However, during diagnostic testing for multicollinearity, we encountered a critical statistical issue: an extremely high correlation between handgrip strength and gender (r = -.959), resulting in Variance Inflation Factor (VIF) values of 12.55 and 12.54, respectively. These values substantially exceeded the recommended threshold of 5 (Craney and Surles 2002; O'Brien 2007), indicating that including both variables would have compromised the reliability and validity of our model.

Following established methodological recommendations (Kim 2019), we chose to exclude handgrip strength from our analyses, as its high correlation with gender made simultaneous inclusion statistically problematic. This decision ensured the consistency and reliability of our regression models. As you recommended, we have now explicitly stated in the Limitations section that, despite our attempt to include handgrip strength, sarcopenia could not be adequately controlled for as a potential confounder.

Figure 1. Correlations (handgrip vs gender)

Figure 2. VIF value

Reference:

Craney, T. A., & Surles, J. G. (2002). Model-Dependent Variance Inflation Factor Cutoff Values. *Quality Engineering*, *14*(3), 391–403. https://doi.org/10.1081/QEN-120001878

O’brien, R.M. A Caution Regarding Rules of Thumb for Variance Inflation Factors. *Qual Quant* **41**, 673–690 (2007). https://doi.org/10.1007/s11135-006-9018-6

Kim, J. H. (2019). Multicollinearity and misleading statistical results. *Korean journal of anesthesiology*, *72*(6), 558-569.

**Reviwer 3:** Recruitment setting and exclusion criteria should be clarified.

**Answer:** The recruitment setting and exclusion criteria are already detailed in the Methods section under "2.1 Study design and setting" and "2.2 Participants and Sample Size". As described, participants were recruited from two nursing homes in Isparta, Turkey, between December 23-30, 2024. A total of 298 residents were assessed for eligibility, and 225 were excluded based on the predefined exclusion criteria listed in the manuscript. Although we recorded the exact numbers excluded for each criterion, we chose not to include these detailed frequencies in the manuscript to avoid lengthening the text unnecessarily. The remaining 73 participants constituted the final sample. We believe this provides a comprehensive and transparent description of the participant selection process. If the editor or reviewers feel that including these detailed exclusion numbers would strengthen the manuscript, we are happy to add them.

**Reviwer 3:** In conclusion, the study demonstrates a statistical association between presbyphagia and urinary incontinence. Older adults classified as having presbyphagia score worse on ICIQ-SF, KHQ, and quality-of-life questionnaires. These findings are compatible with broader constructs such as frailty, comorbidity, and symptom burden. However, the study does not establish a causal relationship, does not identify a shared physiological mechanism, and does not justify clinical screening for urinary incontinence based on presbyphagia alone. The manuscript may be considered for publication provided the above issues are adequately addressed and the exploratory nature of the findings is clearly acknowledged.

**Answer:** We fully agree that this study demonstrates a statistical association rather than a causal relationship. In response, we have emphasized the exploratory nature of our findings throughout the manuscript, replaced all causal language with associative terminology, softened clinical implications, and expanded the Limitations section to address the issues raised. We believe these revisions adequately address the reviewer's concerns.
