# Supplementary material for: The association of presbyphagia with urinary incontinence and Incontinence-related quality of life in nursing home residents: A cross-sectional study
Source: PLoS One. 2026 Jun 4;21(6):e0350933. doi: 10.1371/journal.pone.0350933 (PMC13235875; doi:10.1371/journal.pone.0350933)
Supplement: Attachment — Submitted filename: Response to Reviewers 2.docx [file pone.0350933.s003.docx]

**RESPONCE TO REVİEWERS 1**

**Reviewer 1:** “First, I want to congratulate the authors for their dedication in revising this manuscript. The effort made to effectively improve the work is evident.

The methodological and statistical adjustments made were pertinent and fully addressed my previous assessment…, …. I hope to read this published work soon...”

**Answer:** We sincerely thank the reviewer for their encouraging and generous comments. We are delighted to hear that the methodological and statistical adjustments were considered pertinent and that our revisions have fully addressed the previous concerns. It is a great pleasure to know that the reviewer hopes to read our published work soon. We greatly appreciate their time and constructive input throughout the review process.

**Reviewer 1: “**…There are some minor details throughout the text that appear to be typos and deserve attention (they are indicated in the PDF)...” in PDF: “Typo? The methodology specifies a maximum age of 95 years.”

**Answer:** We thank the reviewer for noting this point. The inclusion criteria specified an age range of 65–95 years; however, the oldest participant in our sample was 92 years. This is not a typo but simply reflects the actual age distribution of the residents who met all inclusion criteria during the study period. We have confirmed that the text is accurate and no correction is needed. We appreciate the reviewer’s careful attention to detail.

**Reviewer 1:** “…I suggest that when citing only one author in a paragraph, the identification should be unique to avoid tiring the reader; therefore, I suggest a careful review of the writing…”

**Answer:** We thank the reviewer for this valuable suggestion. Accordingly, we have revised the two paragraphs concerning De Jesus Fidyk et al. and Matsumoto et al. In each paragraph, the reference is now cited only once at the beginning, with subsequent mentions replaced by pronouns such as "they" or "their study" to avoid unnecessary repetition. We appreciate the reviewer’s careful reading.

**RESPONCE TO REVİEWERS 2**

**Reviewer 2:** “The authors have made a clear effort to address the concerns raised in the previous round of review, and the manuscript has improved in terms of clarity, statistical reporting, and the use of appropriate associative language. The research question is relevant, and the use of validated instruments strengthens the methodological approach.

Overall, the study is methodologically sound, and the analyses are appropriate for the cross-sectional design. The results are clearly presented, and the conclusions are generally supported by the data at the level of association…”

**Answer:** We thank the reviewer for their positive and constructive feedback. We are glad that the revisions have met their expectations.

**Reviewer 2: “**However, a few points would benefit from further clarification and refinement:

* Although some confounders have been considered, there remains the possibility of residual confounding from factors such as sarcopenia, frailty, or overall functional decline. While these are now acknowledged, their potential influence on the observed associations should be more consistently emphasized in the discussion and conclusion…”

**Answer:** We have now more consistently highlighted the potential influence of residual confounding (e.g., sarcopenia, frailty) in both the Discussion and Conclusion sections. However, we deliberately avoided a detailed comparison between these factors and swallowing dysfunction in the Discussion, as it would have disrupted the logical flow of the manuscript and made this section excessively long.

**Reviewer 2:** “*Both the exposure and outcome measures rely on self-reported instruments, which introduces the possibility of shared method bias or general symptom reporting tendencies. This limitation should be highlighted more explicitly.”

**Answer:** We have now explicitly highlighted the possibility of shared method bias and general symptom reporting tendencies in the Limitations section (third point), as suggested. The revised text clarifies that the use of symptom‑based questionnaires for both exposure and outcomes may have inflated the observed associations.

**Reviewer 2:** *Urinary incontinence is treated as a single entity, whereas different subtypes may have distinct underlying mechanisms. This should be more clearly acknowledged as a limitation when interpreting the findings.

**Answer**: We have now elaborated on this limitation in more detail in the Limitations section, explicitly noting that stress and urgency UI may involve distinct pathophysiological mechanisms, which limits the interpretation of our findings. Thank you for the suggestion.

**Reviewer 2:** *The manuscript would benefit from minor language editing to improve clarity and readability, as there are occasional grammatical errors and instances of awkward phrasing.

**Answer:**  The suggestion has been taken into account. The article has been reviewed again and the grammatical errors have been corrected.

**RESPONCE TO REVİEWERS 3**

**Reviewer 3:** “We thank the reviewer for this suggestion. We have now defined "urinary incontinence (UI)" at the very beginning of the Introduction section, before using the abbreviation. The first sentence of the manuscript has been revised accordingly.”

**Answer:** We thank the reviewer for this suggestion. We have now defined "urinary incontinence (UI)" at the very beginning of the Introduction section, before using the abbreviation. The first sentence of the manuscript has been revised accordingly.
